# An automated subtraction of NLO EW infrared divergences

**DOI:** 10.1140/epjc/s10052-018-5600-z

**Published:** 2018-02-09

**Authors:** Marek Schönherr

**Affiliations:** Theoretical Physics Department, CERN, 1211 Geneva 23, Switzerland

## Abstract

In this paper a generalisation of the Catani–Seymour dipole subtraction method to next-to-leading order electroweak calculations is presented. All singularities due to photon and gluon radiation off both massless and massive partons in the presence of both massless and massive spectators are accounted for. Particular attention is paid to the simultaneous subtraction of singularities of both QCD and electroweak origin which are present in the next-to-leading order corrections to processes with more than one perturbative order contributing at Born level. Similarly, embedding non-dipole-like photon splittings in the dipole subtraction scheme discussed. The implementation of the formulated subtraction scheme in the framework of the Sherpa Monte-Carlo event generator, including the restriction of the dipole phase space through the $\alpha$-parameters and expanding its existing subtraction for NLO QCD calculations, is detailed and numerous internal consistency checks validating the obtained results are presented.

## Introduction

As Run-I of the LHC has been successfully completed, culminating in the celebrated experimental confirmation of the existence of the Higgs boson, Run-II proceeds its data-taking at the unprecedented centre-of-mass energy of 13 TeV. As the much anticipated discovery of signals of beyond-the-Standard-Model physics is still lacking, precision tests scrutinising the Standard Model are of prime importance, now and in the foreseeable future. At the same time, new physics searches are looking for increasingly small signals demanding more precise estimates of the Standard Model backgrounds. This expansion of sensitivity of both precision measurements and new physics searches in the multi-TeV region demand an immense improvement of theoretical predictions.

This precision can be achieved by the inclusion of next-to and next-to-next-to-leading order (NLO and NNLO) corrections in the strong coupling and next-to-leading order electroweak (EW) corrections. Here it should be noted that both NNLO QCD and NLO EW corrections are expected to be of a similar magnitude for inclusive observables as numerically $\alpha _s^2\approx \alpha$. On selected differential distributions, however, electroweak corrections can grow much larger. They are dominated by photon emissions in the distributions of final state leptons, for example. In invariant mass spectra of lepton pairs below a resonance, for example, *O*(1) corrections can be present, in which case a proper resummation should be included [1]. Similarly, looking at the (multi-)TeV regime, the NLO EW corrections quickly grow considerably, reducing cross sections by a few tens of percent, due to the emergence of large electroweak Sudakov corrections arising as the scattering energies $Q^2\gg m_W^2$ [2–17]. In this regime they are larger than even the NLO QCD corrections in many cases and their omission becomes the dominant uncertainty in experimental studies and searches.

To this end, benefiting from the well-established techniques developed for the automation of NLO QCD corrections many NLO EW corrections have been calculated recently [18–38]. To fully automate these computations at NLO EW accuracy in a Monte-Carlo framework all infrared divergences need to be regulated, where various incarnations of subtraction methods have proven to be the methods of choice for practical implementations [39–43]. Similar subtractions have also been published for NLO EW calculations using a dipole picture [1, 44–46]. Contrary to the QCD case, only the implementation of [46], restricted to photon emissions of fermions, is publicly available though.

Besides the generalisation to all divergent splittings at $\mathcal {O}(\alpha )$, including photon splittings and photon emissions off massive scalars and vector bosons, this publication addresses the issue of automatically detecting simultaneously occurring QCD and QED singularities and subtracting them consistently. These occur as soon as the Born process is defined at multiple orders $\mathcal {O}(\alpha _s^n\alpha ^{N-n})$. In this case the NLO EW correction to the $\mathcal {O}(\alpha _s^n\alpha ^{N-n})$ process, being of $\mathcal {O}(\alpha _s^n\alpha ^{N-n+1})$, is at the same time the NLO QCD correction to the $\mathcal {O}(\alpha _s^{n-1}\alpha ^{N-n+1})$ process and will in general exhibit the corresponding infrared singularities. Further, matters of the organisation of the contributing partonic processes and their mapping to reduce the computational complexity along with the provision of infrared safe phase space cuts are discussed. The algorithm is implemented in the Amegic [47] matrix element generator which is part of the Sherpa [48] Monte-Carlo event generator framework. It bases on the automated subtraction of massless NLO QCD divergences therein [49, 50]. The implementation presented in this publication has, in various preliminary forms, already been used to calculate electroweak corrections to a multitude of important signal and background processes [19, 21, 25, 31, 32, 34, 37, 51–53], highlighting its versatility.

The present paper is structured as follows: first, in Sect. 2 the Catani–Seymour dipole subtraction method is reviewed and the general modifications to its differential and integrated subtraction terms are discussed. Section 3 then details its automation in Sherpa ’s matrix element generator Amegic, highlighting the necessary changes and improvements with respect to [49, 50]. This section also discusses various options implemented for the incorporation of photon splittings, general infrared safe fiducial phase space definitions and flavour scheme conversions. Essential cross checks validating the presented implementation are then presented in Sect. 4 before concluding in Sect. 5. Explicit formulae for all differential and integrated dipoles are given in Appendix A–C.

**Fig. 1:**
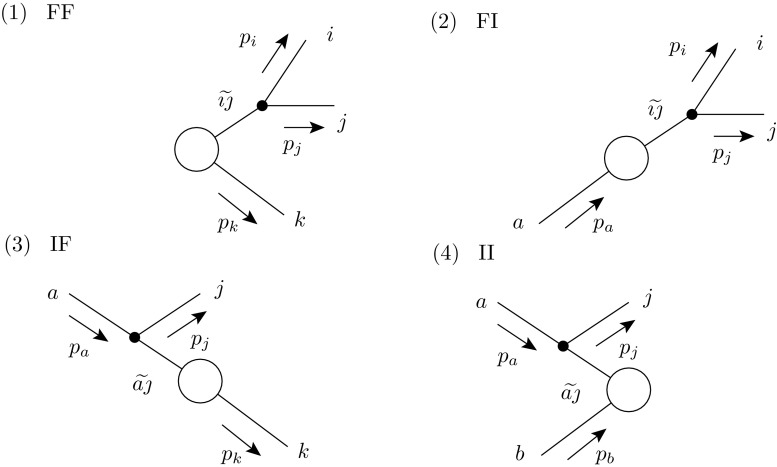
Classification of the four dipole types in Catani–Seymour-type dipole subtraction

## Catani–Seymour subtraction at NLO EW

In order to be applicable to NLO EW calculations the well-known Catani–Seymour dipole subtraction [40, 41] needs to be recast in a suitable form. To highlight the changes from the original formulation for NLO QCD calculations the complete structure of the formalism is reviewed. This subtraction formalism starts from the the expectation value of any infrared safe observable *O* described at NLO accuracy through

2.1 $$\begin{aligned} \langle O\rangle ^\text {NLO}= & {} \int \mathrm{d}\Phi _m^{(4)}\;\mathrm {B}(\Phi _m^{(4)})\,O(\Phi _m^{(4)})\nonumber \\&+\left[ \int \mathrm{d}\Phi _m^{(d)}\; \left[ \mathrm {V}(\Phi _m^{(d)}) +\mathrm {C}(\Phi _m^{(d)}) \right] O(\Phi _m^{(d)})\right. \nonumber \\&\qquad \left. +\int \mathrm{d}\Phi _{m+1}^{(d)}\; \mathrm {R}(\Phi _{m+1}^{(d)})\;O(\Phi _{m+1}^{(d)}) \right] _{\epsilon =0}.\nonumber \\ \end{aligned}$$

Therein, the Born term $\mathrm {B}$ consist of the squared matrix element $\left| \mathcal {M}_m\right| ^2={}_m\langle s_1,\ldots ,s_m|s_1,\ldots ,s_m\rangle {}_m$ with helicity states $s_n$ and further includes all parton densities, parton fluxes, symmetry and averaging factors. The virtual and real corrections, $\mathrm {V}$ and $\mathrm {R}$, as well as the collinear counter term, $\mathrm {C}$, are defined analogously. When regulating their respective divergences through dimensional regularisation, they have to be evaluated consistently in $d=4-2\epsilon$ dimensions for all singularities to cancel. Only after their summation can the limit $\epsilon \rightarrow 0$ be taken. $\mathrm{d}\Phi _n^{(4)}$ and $\mathrm{d}\Phi _n^{(d)}$ are the four and *d* dimensional phase space element. As $\mathrm {V}$ and $\mathrm {C}$ on the one hand side and $\mathrm {R}$ on the other are defined on phase spaces of different parton multiplicity, Eq. (2.1) cannot be used for numerical evaluation straight forwardly and it is rewritten as

2.2 $$\begin{aligned} \langle O\rangle ^\text {NLO}= & {} \int \mathrm{d}\Phi _m^{(4)}\;\mathrm {B}(\Phi _m^{(4)})\,O(\Phi _m^{(4)})\nonumber \\&+\int \mathrm{d}\Phi _m^{(4)}\; \Bigg [ \mathrm {V}(\Phi _m^{(d)}) +\mathrm {C}(\Phi _m^{(d)})\nonumber \\&\quad +\int \mathrm{d}\Phi _1^{(d)}\,\mathrm {D}(\Phi _m^{(d)}\cdot \Phi _1^{(d)}) \Bigg ]_{\epsilon =0} O(\Phi _m^{(4)})\nonumber \\&+\int \mathrm{d}\Phi _{m+1}^{(4)} \left[ \mathrm {R}(\Phi _{m+1}^{(4)})\;O(\Phi _{m+1}^{(4)})\right. \nonumber \\&\quad \left. -\mathrm {D}(\Phi _m^{(4)}\cdot \Phi _1^{(4)})\;O(\Phi _m^{(4)}) \right] , \end{aligned}$$

introducing the subtraction term $\mathrm {D}$. For its construction it is a mandatory requirement that $\mathrm {D}\rightarrow \mathrm {R}$ in all singular limits, rendering the integral on the third line finite in four dimensions. The divergences of the virtual correction and the collinear counterterm on the other side are cancelled separately by the integral of the subtraction term over the one-particle phase space, rendering the integrals of the second line finite in four dimensions as well. Section 2.1 now describes the construction of the differential subtraction term, $\mathrm {D}$, used to subtract all divergences from the real emission correction, while Sect. 2.2 presents the integrated subtraction terms, $\mathrm {I}_\mathrm {D}=\int \mathrm{d}\Phi _1\;\mathrm {D}$, that subtracts all divergences from the virtual corrections and the collinear counterterm in its explicit Laurent expansion after being analytically integrated over the factorised one-particle phase space.

All infrared divergences that occur at NLO EW are of QED origin. No subtractions of potentially large, but finite, corrections involving the emissions of real and virtual massive electroweak gauge bosons will be considered. The practical implementation described in Sect. 3 follows the general lines of [49, 50].

### Differential subtraction terms

To describe all singular limits of a given real emission matrix element $\mathcal {R}$, related to the real emission term of Eq.  (2.1) in a similar fashion as the Born term, it is decomposed into a sum over dipoles $\mathcal {D}$ [40, 41] as

2.3 $$\begin{aligned} \left| \mathcal {M}_{n+1}\right| ^2= & {} \mathcal {R} \,\rightarrow \;\mathcal {D}= \sum \limits _{i,j}\sum \limits _{k\ne i,j}\mathcal {D}_{ij,k} +\sum \limits _{i,j}\sum \limits _{a}\mathcal {D}_{ij}^a \nonumber \\&+\sum \limits _{a,j}\sum \limits _{k\ne j}\mathcal {D}_{j,k}^{a} +\sum \limits _{a,j}\sum \limits _{b\ne a}\mathcal {D}_j^{a,b}. \end{aligned}$$

Therein, *i* is the emitter in the final state, *j* is the emittee, *k* is the spectator in the final state, *a* and *b* are the initial state partons. Each dipole thus encodes the singularity structure caused by the emission of *j* in the presence of the charge of the spectator. While the divergence associated with the soft emission of *j* off the dipole ${\widetilde{\imath \jmath }}-{\tilde{k}}$ is partially fractioned into a piece associated with a splitting ${\widetilde{\imath \jmath }}\rightarrow i+j$ in the presence of spectator *k* and a piece where *i* and *k* swap their roles, the divergence associated with the collinear emission of *j* off ${\widetilde{\imath \jmath }}$ is recovered through charge conservation once all dipoles having ${\widetilde{\imath \jmath }}$ as emitter are summed over.

All four dipole types are depicted in Fig. 1. The individual dipoles take the form [40, 41, 54, 55]

2.4 $$\begin{aligned}&\mathcal {D}_{ij,k} = -\frac{1}{(p_i+p_j)^2-m_{\widetilde{\imath \jmath }}^2}\;{{\varvec{Q}}}_{{\widetilde{\imath \jmath }}{\tilde{k}}}^2\; {}_m\langle \ldots ,{\widetilde{\imath \jmath }},\ldots ,{\tilde{k}}, \nonumber \\&\qquad \qquad \ldots |\mathbf {V}_{ij,k}| \ldots ,{\widetilde{\imath \jmath }},\ldots ,{\tilde{k}},\ldots \rangle {}_m\; \Theta (\alpha _\text { FF}-y_{ij,k}) \nonumber \\&\mathcal {D}_{ij}^a = -\frac{1}{(p_i+p_j)^2-m_{\widetilde{\imath \jmath }}^2}\,\frac{1}{x_{ij,a}} {{\varvec{Q}}}_{{\widetilde{\imath \jmath }}{\tilde{a}}}^2\; {}_m\langle \ldots ,{\widetilde{\imath \jmath }},\ldots ,{\tilde{a}},\nonumber \\&\qquad \qquad \ldots |\mathbf {V}_{ij}^a| \ldots ,{\widetilde{\imath \jmath }},\ldots ,{\tilde{a}},\ldots \rangle {}_m\; \Theta (\alpha _\text { FI}-1+x_{ij,a})\nonumber \\&\mathcal {D}_{j,k}^{a} = -\frac{1}{2p_ap_j}\,\frac{1}{x_{aj,k}}\;{{\varvec{Q}}}_{{\widetilde{a\;\!\!\jmath }}{\tilde{k}}}^2\; {}_m\langle \ldots ,{\widetilde{a\;\!\!\jmath }},\ldots ,{\tilde{k}},\nonumber \\&\qquad \qquad \ldots |\mathbf {V}_{j,k}^a| \ldots ,{\widetilde{a\;\!\!\jmath }},\ldots ,{\tilde{k}},\ldots \rangle {}_m\; \Theta (\alpha _\text { IF}-u_j)\nonumber \\&\mathcal {D}_j^{a,b} = -\frac{1}{2p_ap_j}\,\frac{1}{x_{aj,b}}\;{{\varvec{Q}}}_{{\widetilde{a\;\!\!\jmath }}{\tilde{b}}}^2\; {}_m\langle \ldots ,{\widetilde{a\;\!\!\jmath }},\ldots ,{\tilde{b}},\nonumber \\&\qquad \qquad \ldots |\mathbf {V}_j^{a,b}| \ldots ,{\widetilde{a\;\!\!\jmath }},\ldots ,{\tilde{b}},\ldots \rangle {}_m\; \Theta (\alpha _\text { II}-v_j). \end{aligned}$$

Therein, the charge-correlator is defined as [1, 44, 45]

2.5 $$\begin{aligned} {{\varvec{Q}}}_{{\widetilde{\imath \jmath }}{\tilde{k}}}^2&= \left\{ \begin{array}{ll} \frac{Q_{\widetilde{\imath \jmath }}Q_{\tilde{k}}\theta _{\widetilde{\imath \jmath }}\theta _{\tilde{k}}}{Q_{{\widetilde{\imath \jmath }}}^2} &{} {\widetilde{\imath \jmath }}\ne \gamma \\ \kappa _{{\widetilde{\imath \jmath }}{\tilde{k}}} &{} {\widetilde{\imath \jmath }}=\gamma \end{array}\right. \quad \text {and}\quad \sum _{{\tilde{k}}\ne {\widetilde{\imath \jmath }}}\kappa _{{\widetilde{\imath \jmath }}{\tilde{k}}}=-1\quad \forall {\widetilde{\imath \jmath }}=\gamma . \end{aligned}$$

The $Q_{\widetilde{\imath \jmath }}$ and $Q_{\tilde{k}}$ are the charges of the emitter and the spectator and their $\theta _{{\widetilde{\imath \jmath }}/{\tilde{k}}}$ are $1 (-1)$, if they are in the final (initial) state. Of course, $Q_{\tilde{k}}=Q_k$ and $\theta _{\tilde{k}}=\theta _k$. In the case of photon splittings no soft divergence is present. Thus, these splittings have no dipole character. To include them in the dipole-formalism nonetheless and to distribute the recoil in splittings away from the collinear limit, spectator partons need to be assigned. As their only role is to absorb transverse momentum of the splitting process, any other particle may be considered as spectator. Each thus assigned recoil partner may be assigned a weight $\kappa _{{\widetilde{\imath \jmath }}{\tilde{k}}}$, only their sum is constraint by Eq. (2.5) in order to add up to the correct collinear limit. Various options to assign recoil partners are implemented, they are detailed in Sect. 3.2.

Since the QED charges are real numbers, the charge-correlator simply multiplies the matrix element and only leaves the spin-correlators $\mathbf {V}_{ij,k}$, $\mathbf {V}_{ij}^a$, $\mathbf {V}_{j,k}^a$ and $\mathbf {V}_j^{a,b}$ as insertions in the spin-correlated underlying Born matrix elements. The spin-correlators directly correspond to their QCD counterparts and are detailed in Appendix A. It also defines the initial state momentum rescaling parameters $x_{ij,a}$, $x_{aj,k}$ and $x_{aj,b}$, as well as the splitting variables $y_{ij,k}$, $u_j$ and $v_j$. The $\{\alpha _\text {dip}\}=\{\alpha _\text { FF},\alpha _\text { FI},\alpha _\text { IF},\alpha _\text { II}\}$ parameters serve to restrict the phase space where the individual dipole terms are non-zero and therefore need to be evaluated [54, 55]. They are constructed such that for every $\alpha _{{\widetilde{\imath \jmath }}{\tilde{k}}}>0$ the singularity is fully subtracted. The introduction of a parameter $\kappa$ in dipoles where a final state photon splits into a massive fermion pair in the presence of a final state spectator similarly allows a redistribution of finite terms, cf. Appendix A.

### Integrated subtraction terms

By the above construction, the integral of the subtraction terms $\mathrm {D}$ over the one-particle phase space possesses all the necessary poles to render the second line in Eq. (2.2) finite as $\epsilon \rightarrow 0$. This section now summarises the formulae and findings of [40, 41], translated to the QED case, and discusses their important features. The integrated subtraction terms, together with the collinear counterterm, are commonly reorganised into ${{\varvec{I}}}, {{\varvec{K}}}$ and ${{\varvec{P}}}$ operators through the following identification

2.6 $$\begin{aligned}&\sum _{a,b}\int \mathrm{d}\eta _a\mathrm{d}\eta _b \int \mathrm{d}\Phi _m^{(4)}\; \Bigg [ \mathrm {V}_{ab}(\Phi _m^{(d)})\nonumber \\&\qquad +\,\mathrm {C}_{ab}(\Phi _m^{(d)}) +\int \mathrm{d}\Phi _1^{(d)}\,\mathrm {D}_{ab}(\Phi _m^{(d)}\cdot \Phi _1^{(d)}) \Bigg ]_{\epsilon =0} O(\Phi _m^{(4)}) \nonumber \\&\quad = \sum _{a,b}\int \mathrm{d}\eta _a\mathrm{d}\eta _b \int \mathrm{d}\Phi _m^{(4)}\; \Bigg \{ \left[ \mathrm {V}_{ab}(\Phi _m^{(d)})\right. \nonumber \\&\qquad \quad \left. +\,\mathrm {B}_{ab}(\Phi _m^{(d)})\cdot {{\varvec{I}}}(\epsilon ,\mu ^2;\kappa ,\{\alpha _\text {dip}\}) \right] _{\epsilon =0}\nonumber \\&\qquad +\sum _{a'}\int \mathrm{d}x_a\; \mathrm {B}_{a'b}(\Phi _m^{(4)})\cdot \left[ {{\varvec{K}}}_{aa'}(x_a;\{\alpha _\text {dip}\})\right. \nonumber \\&\qquad \quad \left. +\,{{\varvec{P}}}_{aa'}(x_a;\mu _F^2) \phantom {\Phi _m^{(4)}} \right] \nonumber \\&\qquad +\sum _{b'}\int \mathrm{d}x_b\; \mathrm {B}_{ab'}(\Phi _m^{(4)})\cdot \left[ {{\varvec{K}}}_{bb'}(x_b;\{\alpha _\text {dip}\})\right. \nonumber \\&\qquad \quad \left. +\,{{\varvec{P}}}_{bb'}(x_b;\mu _F^2) \phantom {\Phi _m^{(4)}} \right] \Bigg \}\; O(\Phi _m^{(4)}). \end{aligned}$$

The ${{\varvec{I}}}$ operator contains the necessary infrared poles to cancel all divergences of the virtual correction such that, after summing both, the limit $\epsilon \rightarrow 0$ can be taken and the integral can be evaluated in four dimensions. The ${{\varvec{K}}}$ and ${{\varvec{P}}}$ operators are infrared finite by construction and, thus, evaluation in four dimension is unproblematic as well. Please note, that due to the fact that, contrary to the colour correlator in QCD, the charge correlator in QED is a simple real number and thus the convolution of the Born matrix element with the respective insertion operators in QCD becomes a trivial product of the Born matrix element and the operators in QED. The spin-correlation that was still present in the real subtraction terms has been integrated out in full analogy to the QCD case. In the following, the structure of all three operators in the QED case is discussed.

**The** ${{\varvec{I}}}$ **operator.** The ${{\varvec{I}}}$ operator contains all flavour-diagonal endpoint contributions and cancels all divergences present in the one-loop matrix elements. It takes the general form

2.7 $$\begin{aligned} {{\varvec{I}}}(\epsilon ,\mu ^2;\kappa ,\{\alpha _\text {dip}\})&=-\frac{\alpha }{2\pi }\,\frac{(4\pi )^\epsilon }{\Gamma (1-\epsilon )}\;\nonumber \\&\quad \times \sum _i\sum _{k\ne i}\; {{\varvec{I}}}_{ik}(\epsilon ,\mu ^2;\kappa ,\{\alpha _\text {dip}\}) \end{aligned}$$

and contains single and, in case of massless emitters, double poles in $\epsilon$. Due to the presence of such poles a dependence on the regularisation scale $\mu ^2$ enters. It is commonly identified with the renormalisation scale $\mu _\text {R}^2$. The ${{\varvec{I}}}$ operator is further dependent on the choice of the $\{\alpha _\text {dip}\}$ and $\kappa$ parameters that effect non-singular terms only. It is further decomposed into dipoles [41]

2.8 $$\begin{aligned}&{{\varvec{I}}}_{ik}(\epsilon ,\mu ^2;\kappa ,\{\alpha _\text {dip}\}) \nonumber \\&\quad = {{\varvec{Q}}}_{ik}^2 \left[ \mathcal {V}_{ik}(\epsilon ,\mu ^2;\kappa ) +\Gamma _i(\epsilon ,\mu ^2)+\,\gamma _i\left( 1+\ln \frac{\mu ^2}{s_{ik}}\right) \right. \nonumber \\&\qquad \qquad \qquad \left. +\,K_i+A_{ik}^I(\{\alpha _\text {dip}\}) +\mathcal {O}(\epsilon ) \right] ,\end{aligned}$$

wherein *i* labels the emitter and *k* the spectator. The charge insertion operator, which is a trivial real number in the QED case, is defined in Eq. (2.5). The full crossing invariance of the QCD ${{\varvec{I}}}$ operator may be broken in the QED case in the presence of photon splittings as their recoil partner assignment is arbitrary and may involve information on initial or final state particles. Different possible choices are discussed in Sect. 3.2, some of which may break this crossing invariance.

The divergences of the ${{\varvec{I}}}$ operator are encoded in the functions $\mathcal {V}_{ik}$ and $\Gamma _i$. While the former contains all soft-(quasi-)collinear divergences the latter contains the pure (quasi-)collinear ones. They do not only differentiate whether the emitter is a photon or not, but also between different spins of the emitter. Their precise form as well as the the flavour constants $\gamma _i$ and $K_i$ are given in Appendix B. $A_{ik}^I$ encodes the dependence on the phase space restriction of the individual dipoles $\{\alpha _\text {dip}\}$. Finite terms originating in dipoles involving initial state legs, however, can be pushed into the ${{\varvec{K}}}$ operator. Thus, $A_{ik}^I$ by convention only depends on $\alpha _\text { FF}$. Its precise form is given in Appendix C.

**The** ${{\varvec{K}}}$ **and** ${{\varvec{P}}}$ **operators.** The ${{\varvec{K}}}$ and ${{\varvec{P}}}$ operators collect all pieces of the integrated dipole terms that are not collected in the ${{\varvec{I}}}$ operator and combines them with the collinear counterterms $\mathrm {C}$ to give a finite result as $\epsilon \rightarrow 0$. By construction they contain only remainders of splittings where either the emitter or the spectator is in the initial state. Thus, they are comprised of terms arising due to the change of the flavour or the partonic momentum fraction *x* of an initial state due to a splitting.

The ${{\varvec{K}}}$-operator is given by [41]2.9It depends on the partonic *x*, and the flavour change from the Born process initial state flavour *a* to $a'$ of the convolution Eq. (2.6). Therein, the $\overline{K}$ collect universal terms present in all splitting involving an initial state as either emitter or spectator. Then, while $\mathcal {K}$ contains solely remaining terms from final state splittings in the presence of initial state spectators, the $K_{}^t$ are their counterparts for initial state splittings in the presence of a final state spectator, *i* and *k* running over all final state partons in each case. $\tilde{K}$ contains solely related correlations between both initial states, arising from dipoles where both the emitter and the spectator are in the initial state. The $A^K$ terms collect all finite terms arising when any of $\alpha _\text { FI}$, $\alpha _\text { IF}$ or $\alpha _\text { II}$ is different from unity, thus restricting the phase space of the respective dipoles. Again, the ${{\varvec{Q}}}_{ik}^2$ are the charge correlators of Eq. (2.5). Finally, $K_\text {FS}$ contains the factorisation scheme dependence. Currently, both only the $\overline{\text {MS}}$ schemes is supported, setting these terms identically zero.

The ${{\varvec{P}}}$-operator now collects the remaining initial state collinear singularity from all dipoles involving initial states either as emitters or as spectators and cancels them against the collinear counterterm. Through this counterterm a dependence on the factorisation scale enters. The ${{\varvec{P}}}$ operator is given by [40, 41]

2.10 $$\begin{aligned}&{{\varvec{P}}}_{aa'}(x,\mu _F^2)\nonumber \\&\quad = \frac{\alpha }{2\pi }\;P^{aa'}(x) \left[ \sum \limits _k{{\varvec{Q}}}_{a'k}^2\,\log \frac{\mu _\text {F}^2}{xs_{ak}}+{{\varvec{Q}}}_{a'b}^2\,\log \frac{\mu _\text {F}^2}{xs_{ab}} \right] . \end{aligned}$$

Only initial state splittings are present, either in the presence of a spectator in the final state, which is encoded in the sum of *k*, or with the opposite initial state *b* acting as the spectator. It otherwise only depends on the Alterelli–Parisi splitting function detailed in Appendix B.

## Implementation

The implementation of the QED generalisation of the Catani–Seymour dipole subtraction scheme in Sherpa ’s matrix element generator Amegic proceeds along the lines of [49]. As in general real and virtual corrections of $O(\alpha _s^n\alpha ^m)$ contain divergences of both QCD and QED origin, both cases are included in this section. In the following, the general structure of the implementation is reviewed.

### Identification of dipoles

The starting point to construct the involved subtraction terms in the Catani–Seymour subtraction formalism is a given flavour configuration in the Born or the real emission phase space and the perturbative order $\mathcal {O}(\alpha _s^n\alpha ^m)$ in accordance with the respective virtual or real correction to be computed. For all parts, on-the-fly variations of both the factorisation scale $\mu _\text {F}$ and the renormalisation scale $\mu _\text {R}$ are available through an extension of the algorithm detailed in [56].

**Differential subtraction terms.** A given real emission configuration $\{ab\}\rightarrow \{1,..,m+1\}$ at order $O(\alpha _s^n\alpha ^m)$ can in general exhibit both QCD and QED divergences simultaneously. The following therefore describes the identification of both types of dipoles. Thus, all triplets $\{i,j,k\}$ that can be built from the external particles of the process are tested for the presence of an infrared divergence, QCD or QED, by checking for the existence of a dipole subtraction term. In these triplets *i* and *k* may be in the initial or final state while *j* may be in the final state only. Likewise, $i\ne j$, $i\ne k$, $j\ne k$ and triplets that only differ in a permutation of *i* and *j* are considered identical. Then, the following steps are executed.

1. Based on the quantum numbers and flavours of the triplet it is decided whether a QCD, a QED or both splitting function can exist. A QCD splitting function can exist only if *i*, *j* and *k* are colour charged, while a QED splitting function can exist only if the charge-correlator ${{\varvec{Q}}}_{{\widetilde{\imath \jmath }}{\tilde{k}}}^2$ does not vanish. The dipole type is determined based on whether *i* and *k* are in the initial or final state. A given triplet $\{i,j,k\}$ may exhibit both QCD and QED divergences, and thus may form both a QCD and a QED dipole.
2. The flavours ${\widetilde{\imath \jmath }}$ and ${\tilde{k}}$ are determined for each possible splitting function.
3. For each possible splitting function $\{{\widetilde{\imath \jmath }},{\tilde{k}}\}\rightarrow \{i,j,k\}$ the underlying Born configuration and its order, $O(\alpha _s^{n-1}\alpha ^m)$ in case of a QCD splitting function and $O(\alpha _s^n\alpha ^{m-1})$ in case of a QED splitting function, are determined. If, including the insertion of the appropriate colour-, charge- and spin-correlations, such a process at this order exists, a dipole subtraction term is built.

If the above steps do not lead to a valid dipole subtraction term, no divergence can be present. The real emission, a conventional tree-level process, is grouped together with all its subtraction terms into one computational unit and their respective cross sections and observable values, $O(\Phi _{m+1})$ and all $O(\Phi _{m_i})$, are treated as correlated.

**Integrated subtraction terms.** Similar to the above discussed real emission corrections, the virtual correction configurations $\{ab\}\rightarrow \{1,\ldots ,m\}$ at order $O(\alpha _s^n\alpha ^m)$ in general exhibits poles due to both QCD and QED origins. To subtract them, both QCD and QED integrated subtraction need to be included. In fact, they naturally arise as counterparts to differential subtraction terms constructed for the corresponding real emission correction, as guaranteed by Bloch and Nordsieck [57] or Kinoshita–Lee–Nauenberg [58, 59] theorem. Consequently, QCD and QED ${{\varvec{I}}}, {{\varvec{K}}}$ and ${{\varvec{P}}}$ operators are constructed. While their QCD version are described in detail in [49, 50], their QED version of Eq. (2.6) are discussed below.

The ${{\varvec{I}}}$ operator, on the one hand side, has the same initial state flavours and momentum fractions as the virtual correction. The ${{\varvec{K}}}$ and ${{\varvec{P}}}$ operators on the other hand, resulting from the combination of the integrated subtraction terms and the collinear counterterms, involve a summation over possible initial state flavours and comprise the following general structure in their dependence on the additional *x* integration variable

3.1 $$\begin{aligned} \left[ g(x)\right] _++\delta (1-x)h(x)+k(x). \end{aligned}$$

Therein, both *h*(*x*) and *k*(*x*) are regular functions in *x*, while the plus distribution of *g*(*x*) is defined as

3.2 $$\begin{aligned} \int \limits _0^1\mathrm{d}x\;f(x)\left[ g(x)\right] _+&= \int \limits _0^1\mathrm{d}x\;\left[ f(x)-f(1)\right] g(x). \end{aligned}$$

Hence, the potentially computationally intensive matrix elements in $\mathrm {B}$ have to be calculated twice for every phase space point in addition to the flavour summation. To remedy this, the original expression of Eq. (2.6) is reformulated. Dropping the dependence on the $\{\alpha _\text {dip}\}$ parameters and explicitly stating the dependence of the underlying Born term on the initial state momentum fractions and parton densities, $\mathrm {B}_{ab}=f_af_b\,\mathcal {B}_{ab}$, the ${{\varvec{K}}}$ and ${{\varvec{P}}}$ operators it can be recast to

3.3 $$\begin{aligned}&\sum _{a,b}\int \mathrm{d}\eta _a\mathrm{d}\eta _b \int \mathrm{d}\Phi _m^{(4)}\; \sum _{a'}\int \limits _0^1\mathrm{d}x_a\; \mathrm {B}_{a'b}(x_a\eta _a,\eta _b;\Phi _m^{(4)})\nonumber \\&\qquad \cdot \left[ {{\varvec{K}}}_{aa'}(x_a;\{\alpha _\text {dip}\}) +{{\varvec{P}}}_{aa'}(x_a;\mu _F^2) \phantom {\Phi _m^{(4)}} \right] \;O(\Phi _m^{(4)})\nonumber \\&\quad = \sum _{a,b}\int \mathrm{d}\eta _a\mathrm{d}\eta _b \int \mathrm{d}\Phi _m^{(4)}\; \sum _{a'}\int \limits _0^1\mathrm{d}x_a \Bigg \{ g^{aa'}(x_a) \nonumber \\&\qquad \times \left. \left[ \mathrm {B}_{a'b}(x_a\eta _a,\eta _b;\Phi _m^{(4)}) -\mathrm {B}_{a'b}(\eta _a,\eta _b;\Phi _m^{(4)}) \right] \phantom {\int } \right. \nonumber \\&\qquad \qquad \left. +\,k^{aa'}(x_a)\, \mathrm {B}_{a'b}(x_a\eta _a,\eta _b;\Phi _m^{(4)})\right. \nonumber \\&\qquad \qquad +h^{aa'}(1)\,\mathrm {B}_{a'b}(\eta _a,\eta _b;\Phi _m^{(4)})\phantom {\int } \Bigg \} \;O(\Phi _m^{(4)})\nonumber \\&\quad = \sum _{a,b}\int \mathrm{d}\eta _a\mathrm{d}\eta _b \int \mathrm{d}\Phi _m^{(4)}\; f_b(\eta _b)\, \mathcal {B}_{ab}(\Phi _m^{(4)})\nonumber \\&\qquad \times \sum _{a'} \left\{ \; \int \limits _{\eta _a}^1\mathrm{d}x_a \left[ \tfrac{1}{x_a}\,f_{a'}\!\!\left( \tfrac{\eta _a}{x_a}\right) \left( g^{aa'}(x_a)+k^{aa'}(x_a)\right) \right. \right. \nonumber \\&\left. \left. \qquad \qquad \qquad -f_{a'}(\eta _a)\,g^{aa'}(x_a) \right] \right. \nonumber \\&\qquad \qquad \qquad \left. \phantom {\frac{f_{a'}\left( \frac{\eta _a}{x_a}\right) }{x_af_a(\eta _a)}} +f_{a'}(\eta _a) \left( h^{aa'}-G^{aa'}(\eta _a) \right) \right\} \;O(\Phi _m^{(4)}), \end{aligned}$$

with $f_a(\eta )$ being the parton density of flavour *a* and momentum fraction $\eta$ in the proton, otherwise absorbed in the Born term $\mathrm {B}$. All PDFs are evaluated at the same scale $\mu _\text {F}$. $G^{ab}(\eta )=\int _0^\eta \mathrm{d}x\;g^{ab}(x)$ are the analytically computed divergence free parts of the integral of the functions under the plus distribution. Its divergence at $x=1$ is cancelled numerically on the second last line. Effectively, this reformulation results in a redefinition of the PDF for incoming parton *a* of $\mathrm {B}_{ab}$. As the integrand of the remaining integral over $x_a$ is a simple one-dimensional function without a pronounced structure, its numerical evaluation is very stable and can be accomplished by a single point for each summand per phase space point $\mathrm{d}\Phi _m^{(4)}$. The ${{\varvec{K}}}$ and ${{\varvec{P}}}$ operators for the second incoming parton *b* are recast similarly.

The thus transformed form of the ${{\varvec{K}}}$ and ${{\varvec{P}}}$ operators require only a single evaluation of the potentially costly matrix elements in $\mathrm {B}$ while retaining the number of computations of PDFs needed, speeding up the computation considerably for involved processes. This allows to generate and evaluate the underlying Born matrix element for both the ${{\varvec{I}}}$ operators and the ${{\varvec{K}}}$ and ${{\varvec{P}}}$ operators at the same time. In fact, due to these three operators being simple multiplicative scalars, the common underlying Born matrix element is identical to the standard Born matrix element, allowing for their simultaneous calculation at no extra cost. The operators themselves are then built from dipoles constructed from all doublets $\{i,k\}$, $i\ne k$, of external partons available in the partonic process for which the charge-correlator ${{\varvec{Q}}}_{ik}^2$ does not vanish.

### External photons

External photons can play different roles in a calculation: they can either be resolved or unresolved. According to this distinction they should be treated differently at NLO EW [31, 60]. Initial state photons are always unresolved at a hadron collider. They thus should be treated in a short-distance scheme, allowing them to split into massless fermions, necessitating a proper subtraction of infrared divergences. Final state photons on the other hand can play both roles. If they are considered resolved, they should be treated in an on-shell scheme and no explicit photon splitting is allowed. Concerning the dipole subtraction discussed in this paper they are thus neutral particles and do not form part of a dipole, except as possible recoil partner of another unresolved photon. A final state unresolved photon, on the other hand, again must be treated in a short-distance scheme, necessitating their splittings to be subtracted.

As discussed in Sect. 2.1, the dipole picture is not necessary to capture the divergences of photons splitting in pairs of massless fermions, leptons and quarks, due to the absence of soft divergences that necessitate the correlation with the emissions off other partons of the event. Nonetheless, it offers a possibility to assign one or more recoil partners to absorb the transverse momentum of the splitting, thus fitting these purely collinear splittings into the dipole picture. The choice of spectator is essentially arbitrary, and all other partons of the event offer being viable spectators. Only the following condition has to hold

3.4 $$\begin{aligned} \sum _{{\tilde{k}}\ne {\widetilde{\imath \jmath }}}\kappa _{{\widetilde{\imath \jmath }}{\tilde{k}}} \,=\;-1 \end{aligned}$$

for every splitting photon ${\widetilde{\imath \jmath }}$, cf. 2.5. Therein, the $\kappa _{{\widetilde{\imath \jmath }}{\tilde{k}}}$ are arbitrary and possibly dynamic weights assigned to every dipole with spectator ${\tilde{k}}$. In practise, for initial state as well as final state splittings five choices $c_{\tilde{k}}^\gamma$ have been implemented:

0. only allow initial state partons as spectators,
1. only allow final state partons as spectators,
2. only allow QED charged particles as spectators,
3. only allow QED neutral particles as spectators,
4. allow all particles as spectators.

The $\kappa _{{\widetilde{\imath \jmath }}{\tilde{k}}}$ are set to the phase space point independent value $-n_\text {spec}^{-1}$, with $n_\text {spec}$ the number of assigned spectators, thus trivially fulfilling Eq. (3.4).

### Process mappings

In general, physical cross sections include multiple different partonic channels. However, many of these partonic channels share identical squared matrix elements, potentially differing by constant factors. They, thus, do not need to be recomputed for every flavour channel but can be reused. As a typical example, consider the production of a lepton pair in association with two jets. The process $g\,d\rightarrow e^+e^-\,g\,d$ shares a common squared matrix element with $g\,s\rightarrow e^+e^-\,g\,s$ and $g\,b\rightarrow e^+e^-\,g\,b$ on Born level at $\mathcal {O}(\alpha _s^2\alpha ^2)$. Hence, the squared matrix elements of the latter two partonic processes are mapped on the first, reusing its computed value. In this way, of the 95 partonic channels of this process, only 30 have to be computed. Further mappings of individual graphs and subgraphs are implemented but are not discussed in the following, see [47, 48].

In Sherpa ’s matrix element generators various forms of process mappings are implemented to reduce the computational complexity and memory footprint, both for the virtual and real emission corrections, cf. [47–50]. However, while for NLO QCD corrections to the leading order Born process it is true that if the Born process is mappable onto another existing process, then also both the virtual correction and the insertion-operator-augmented colour-correlated underlying Born process of the integrated subtraction term are mappable to the same process. This is no longer true when considering the NLO EW or NLO QCD corrections to subleading Born processes.

In the present implementation of a full NLO QCD and NLO EW subtraction in the matrix element generator Amegic the following process mapping strategy is followed for the real subtracted contributions:

- The real emission process and its associated dipole subtraction terms are grouped in one computational unit. A given partonic channel of the real emission process can be mapped onto another already existing one if both processes consist of the same diagrams and all involved (internal and external) particles have the same masses and widths and the same underlying interaction (coupling factors may differ by a constant). Is this the case the whole computational unit can be mapped and the result of the mapped-to process can simply be reused.
- Individual dipoles can be mapped if the emitter, emittee and spectator indices are identical, and the underlying Born process can be mapped according to the above rules. In this case, the result of the mapped-to process can simply be reused.
- Underlying Born matrix elements can be mapped if the Born-level emitter ${\widetilde{\imath \jmath }}$ carries the same indices and the underlying Born process itself can be mapped. The spin correlation insertion operator, needed if parton ${\widetilde{\imath \jmath }}$ is either a gluon or a photon and described in [49], is encoded in the calculational routines and necessitates the above restriction. Can the underlying Born process be mapped, only the calculational routines can be shared, reducing the memory footprint, but due to the potentially differing underlying Born momenta its result has to be recomputed.

The strategy for the virtual subtracted contributions reads as follows:

- The virtual correction process, interfaced from an external virtual correction provider, as well as its associated integrated dipole subtraction terms and the collinear counterterm, taken together and reformulated according to Sect. 3.1, are grouped into one computational unit. If the underlying Born processes of the integrated subtraction contribution (in all orders required) can be mapped according to the above rules and the virtual correction provider confirms that the virtual correction can be mapped onto the same process that Sherpa ’s tree-level matrix element generator maps the underlying Born processes onto, the whole computational unit is mapped. In this case, the result of the mapped-to process can simply be reused. The ${{\varvec{K}}}$ and ${{\varvec{P}}}$ operators, whose internal PDF factors depend on the initial state flavour, still have to be recomputed, but their matrix element coefficients are cached.
- If the virtual correction provider cannot confirm the mapping performed for the underlying Born process, only the underlying Born process is mapped and virtual correction is recomputed. Again, the ${{\varvec{K}}}$ and ${{\varvec{P}}}$ have to be recomputed, but their matrix element coefficients are cached. Here, efficiency is lost if the virtual correction provider uses less efficient process mappings.

### Fiducial phase space definition

Phase space restrictions are an essential part of the implementation of a framework for automated NLO calculcations. These cuts, however, need to be applied in an infrared safe way. At NLO, they must not discriminate between a massless parton before and after its collinear splitting or before and after a soft gluon or photon emission. Thus, if QCD singularities are present, massless QCD charged particles must be clustered into jets before any further cuts are applied. Similarly, in case of the presence of QED divergences, massless charged particles must either be dressed with the surrounding photons or be included in the jet algorithm. Subtleties arise in the presence of both QCD and QED singularities simultaneously. Here, usually, only a fully democratic jet finding can consistently treat all singularities, although specialised solutions exist for simplified situations. Massive QCD and QED charged particles may be treated as bare as their mass shields the collinear singularity, but can also be included into jet finding and dressing algorithms. An intermediate scheme which includes only the logarithms of the parton mass needed to regulate the collinear divergences [45], but are otherwise treated massless in the calculation, is not implemented.

To this end, the implementation in Sherpa is equipped with a range of algorithms to define infrared safe quantities on which further restrictions can be applied. Multiple such selectors can be nested.

- DressedParticleSelector This selector takes a choice of dressing algorithm (cone or sequential recombination) and (flavour-dependent) dressing parameters (cone radius or radial parameter and exponent). All charged particles of the process are then dressed with all photons using the specified algorithm with the given (flavour-dependent) parameters. Their four momenta are added such that four momentum is conserved. The dressed charged particles may no longer be on-shell and the photons used to dress the charged particles are removed from the list of particles. The resulting list of particles and their momenta are then passed to all subselectors.
- Jet_Selector This selector uses FastJet [61] to build jets from a given list of input particles. It takes a list of flavours that are considered as jet finding input particles, the jet finding algorithm and its parameters including phase space boundaries in $p_\perp$, $\eta$ or *y*, as well as a minimal and maximal number of jets to be found as arguments. Additionally, clustered jets can be tagged or anti-tagged based on their flavour content, including relative and absolute constituent momentum requirements (e.g. *b* tagging a jet if one of its constituents is a *b*-quark or anti-$\gamma$-tagging a jet if its photon constitents carry in excess of $z_\text {thr}$ fraction of the total jet momentum). The clustered jets as well as all particles not used as jet finding input are passed on to all subselectors.
- Isolation_Selector This selector uses the smooth cone isolation of [62] to isolate particles of a given flavour against particles of another flavour. It takes both the isolation flavour and rejection flavour list as well as the algorithm parameters as input. It further can be specified how many isolated particles of the given flavour should minimally and maximally be found in a given phase space volume (bounded by $p_\perp$ and $\eta$ or *y* ranges), e.g. exactly two isolated photons with $p_\perp >30\,\text {GeV}$ and $|\eta |<2.35$. The list of found isolated flavours and all other particles except for those that should be isolated, but are not, are passed to all subselectors.

Once an infrared safe definition of particles is found, the standard single- or multi-particle selectors such as PT (implementing transverse momentum requirements on a given flavour), PTmis (missing transverse momentum build from all neutrinos), or DR (angular distance between the two given particles) can be used. Further, Sherpa is equipped with a user hook system, providing the possibility for users to implement arbitrary routines for phase space cuts and dynamically load them at run time, without the need to modify their Sherpa installation.

Identified particles should in principle be defined using fragmentation functions, denoted $D_i^j(z)$ for finding parton *i* in parton *j* at momentum fraction *z*. As, however, all $D_i^i(z)$ have a $\delta (1-z)$ as leading term in on-shell renormalisation schemes (and only differ by ratios of couplings in other schemes) simplified schemes exist that are applicable to many practical situations. Hence, no fragmentation function is implemented yet.

### Flavour scheme conversion

All publicly available PDF sets containing QED effects in their evolution are fitted with five (Mrstqed [63], CT14qed [64], Luxqed [65, 66], HKR16 [67], Nnpdf3.0qed [68], Nnpdf3.1luxqed [69]) or six (Nnpdf2.3qed [70]) light flavours. Thus, for next-to-leading order calculations with four or less light flavours the following scheme-conversion terms need to be added for consistency [71]

3.5 $$\begin{aligned}&\langle O\rangle _\text {NLO QCD}^{({n_\text {lf}})}(\mu _\text {R}^2,\mu _\text {F}^2)\nonumber \\&\quad =\langle O\rangle _\text {NLO QCD}^{({n_\text {f}})}(\mu _\text {R}^2,\mu _\text {F}^2)+ \int \!\mathrm{d}\Phi _m \sum \limits _{i={n_\text {lf}}}^{n_\text {f}}\sum _{\{ab\}} \frac{\alpha _s}{3\pi }\, {T_R}\nonumber \\&\qquad \times \left[ p\,\log \frac{m_i^2}{\mu _\text {R}^2}\,\Theta \left( \mu _\text {R}^2-m_i^2\right) -\Delta _{ab}^{gg}\log \frac{m_i^2}{\mu _\text {F}^2}\, \right. \nonumber \\&\quad \qquad \qquad \left. \Theta \left( \mu _\text {F}^2-m_i^2\right) \right] \mathrm {B}_{ab}^{({n_\text {f}})}(\Phi _m;\mu _\text {R}^2,\mu _\text {F}^2)\;O(\Phi _m) \end{aligned}$$

and

3.6 $$\begin{aligned}&\langle O\rangle _\text {NLO EW}^{({n_\text {lf}})}(\mu _\text {R}^2,\mu _\text {F}^2)\nonumber \\&\quad = \langle O\rangle _\text {NLO EW}^{({n_\text {f}})}(\mu _\text {R}^2,\mu _\text {F}^2)\nonumber \\&\qquad - \int \mathrm{d}\Phi _m \sum \limits _{i={n_\text {lf}}}^{n_\text {f}}\sum _{\{ab\}} \frac{\alpha }{3\pi } {N_C}\,Q_i^2\; \Delta _{ab}^{\gamma \gamma }\log \frac{m_i^2}{\mu _\text {F}^2}\nonumber \\&\qquad \quad \times \Theta \left( \mu _\text {F}^2-m_i^2\right) \; \mathrm {B}_{ab}^{({n_\text {f}})}(\Phi _m;\mu _\text {R}^2,\mu _\text {F}^2)\;O(\Phi _m). \end{aligned}$$

Therein, the $\langle O\rangle _\text {NLO QCD/EW}^{(n)}$ is the expectation value of an arbitrary observable *O* computed at NLO QCD or NLO EW, respectively, with *n*-flavour scheme parton densities and the corresponding strong coupling summed over all initial state contributions. $\mathrm {B}_{ab}^{(n)}$ is the Born term, as defined in Sect. 2, in the *ab* channel. The sums run over all $({n_\text {f}}-{n_\text {lf}})$ flavours of mass $m_i$ that are part of the $n_\text {f}$-flavour PDF parametrisation but not the $n_\text {lf}$-flavour scheme of the calculation and all combinations of partonic channels *ab* occurring in the $n_\text {lf}$-flavour scheme, respectively. *p* is the power of the strong coupling in the Born process and $\Delta _{ab}^{gg}=\delta _{ga}+\delta _{gb}$, i.e. it takes the values 2 in the *gg* channel, 1 in all *qg* and $\bar{q}g$ channels, and zero otherwise. $\Delta _{ab}^{\gamma \gamma }$ is defined analogously. Each logarithm of course only contributes if the scale is larger than the respective mass. As the electroweak coupling is not taken running in the common renormalisation schemes it is independent of the number of massless flavours in the calculation. For $\overline{\text {MS}}$-like renormalisation schemes a similar term proportional to its power in the Born process is to be added.

## Checks of the implementation

The implementation of the formalism described in the previous sections in Sherpa needs to be validated. To this end, both its independence of its internal free parameters, $\{\alpha _\text {dip}\}=\{\alpha _\text { FF},\alpha _\text { FI},\alpha _\text { IF},\alpha _\text { II}\}$, $\kappa$ and the choice of spectator in photon splittings, and its agreement with independent implementations for fixed values of these parameters need to be tested. While the latter were carried out in [19, 21, 25, 31, 32] against the private implementations in Munich [74], in [51] against MadGraph5 [75], and in [51, 53] against Recola [76] and found full agreement, the former represent a powerful check of internal consistency. Further, as the $\{\alpha _\text {dip}\}$ parameters regulate the phase space coverage of the differential subtraction terms, they can be used to lower the average number of contributing dipoles for a given real emission contribution and, thus, reduce the computational costs of the real-subtracted contribution.

There are now two independent aspects of the calculation that need to be checked:

- The ${{\varvec{I}}}$ operator of Sect. 2.2 containing the explicit Laurent expansion in $\epsilon$ as $\epsilon \rightarrow 0$ needs to reproduce the correct $\epsilon ^{-2}$ and $\epsilon ^{-1}$ coefficients in order to cancel all corresponding poles of the virtual matrix elements, leading to a finite integrand in Eq. (2.2) in $d=4$. These checks were performed for all possible dipoles in [19, 21, 25, 31, 32, 34, 37, 51–53] and are not repeated here.
- The expectation value of any infrared observable is independent of the choice of the technical $\{\alpha _\text {dip}\}$ and $\kappa$ parameters as well as the choice $c_{\tilde{k}}^\gamma$ of spectator for photon splittings. It thus needs to be verified that the sum of the contributions of the $\epsilon ^0$ coefficient of the integrated subtraction terms and the differential subtraction terms is independent of these parameters and choices. In order to arrive at a finite result in $d=4$, cf. Eq. (2.2), the real emission correction and the collinear counterterms are added. The corresponding quantity is defined as
  4.1 $$\begin{aligned} \langle O\rangle _\text {IRD}&= \int \mathrm{d}\Phi _m^{(4)}\; \left[ \mathrm {I}_\mathrm {D}(\Phi _m^{(4)};\{\alpha _\text {dip}\},\kappa ,c_{\tilde{k}}^\gamma )\right. \nonumber \\&\qquad \left. +\,\mathrm {C}(\Phi _m^{(4)}) \right] _{\epsilon ^0\,\text {coeff.}} O(\Phi _m^{(4)})\nonumber \\&\quad +\int \mathrm{d}\Phi _{m+1}^{(4)} \left[ \mathrm {R}(\Phi _{m+1}^{(4)})\;O(\Phi _{m+1}^{(4)})\right. \nonumber \\&\qquad \left. -\,\mathrm {D}(\Phi _m^{(4)}\cdot \Phi _1^{(4)};\{\alpha _\text {dip}\},\kappa ,c_{\tilde{k}}^\gamma )\;O(\Phi _m^{(4)}) \right] , \end{aligned}$$
  and will be evaluated for processes containing all available dipole configurations in the following.

In the following, the inclusive or fiducial cross section contribution $\sigma _\text {IRD}$ is evaluated for a range of different processes, testing all possible dipole configurations. Throughout the input parameters of Table 1 are used in the $G_\mu$ scheme, although several other EW input parameter schemes are available in general. Similarly, all unstable particles are treated in the complex-mass scheme [77]. Further, the CT14nlo [78] and CT14qed [64]^1^ PDF sets with five active and massless flavours and their corresponding $\alpha _s$ parametrisations with $\alpha _s(m_Z)=0.118$ are used. While the use of the CT14nlo PDF set for NLO EW calculations is, strictly speaking, inconsistent due its missing QED evolution of the initial state quarks, it offers the quantification of the importance of photon initiated processes in these technical comparisons. To the same end, the CT14qed PDF set is not employed using its the best fit value for the intrinsic inelastic photon momentum fraction at the reference scale of $Q=1.295\,\text {GeV}$ $p_0^\gamma =0.05\%$, but rather evaluate it for the extremes of its $1\sigma$ uncertainty, $p_0^\gamma =0$ and $p_0^\gamma =0.14\%$, where applicable.

**Table 1:** Numerical values of all input parameters. The gauge boson masses are taken from [72], while their widths are obtained from state-of the art calculations. The Higgs mass and width are taken from [73]. The top quark mass is taken from [72] while its width has been calculated at NLO QCD. In calculations where a massive particle is present as an external state, its width is set to zero

$$G_\mu =1.1663787\times 10^{-5}~\text {GeV}^2$$	
$m_W=80.385~\text {GeV}$	$\Gamma _W=2.0897~\text {GeV}$
$m_Z=91.1876~\text {GeV}$	$\Gamma _Z=2.4955~\text {GeV}$
$m_t=173.2~\text {GeV}$	$\Gamma _t=1.339~\text {GeV}$

In processes where jets need to be constructed to define a fiducial phase space volume, anti-$k_t$ jets with $R=0.4$ and $p_\perp >30\,\text {GeV}$ are used [80] and both partons and photons are considered as constituents. Similarly, if leptons need to be defined for the same purpose, they are dressed with all photons in a cone of $\Delta R=0.1$. The number of phase space points in the computation of $\sigma _\text {IRD}(\{\alpha _\text {dip}\},\kappa ,c_{\tilde{k}}^\gamma )$ is kept constant for each process, such that the indicated statistical uncertainty can be interpreted as a measure of the change of convergence of the subtraction with respect to the variation of the technical parameter choices.

**Fig. 2:**
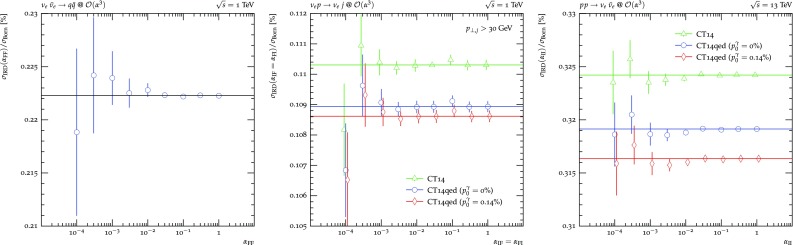
$\{\alpha _\text {dip}\}$-dependence of the sum of integrated subtraction term and differentially subtracted real emission for $\nu _e\bar{\nu }_e\rightarrow jj$, $\nu _e p\rightarrow \nu _e j$ and $pp\rightarrow \nu _e\bar{\nu }_e$

**Massless dipoles.** Contrary to the QCD case, in the Standard Model almost all particles carry QED charges and therefore participate in the construction of dipoles. One notable exception are neutrinos. Therefore, in order to investigate the behaviour of the massless II, IF, FI and FF dipoles, and, thus, the independence of $\alpha _\text { II}, \alpha _\text { IF}, \alpha _\text { FI}$ and $\alpha _\text { FF}$ separately in this technical validation $\sigma _\text {IRD}$ is considered for all three different rotations of the interaction of a quark-anti-quark pair with a neutrino-anti-neutrino pair. Hence, besides the $\sigma _\text {IRD}$ contribution to the $\mathcal {O}(\alpha )$ correction to $pp\rightarrow \nu _e\bar{\nu }_e$ at the LHC at an invariant mass of 13 TeV, $\sigma _\text {IRD}$ is computed for both the production of at least two jets at a hypothetical $\nu _e-\bar{\nu }_e$ collider at a centre-of-mass energy of 1 TeV and inclusive single jet production in equally hypothetical $\nu _ep$ deep inelastic scattering (DIS) with the same centre-of-mass energy are calculated.

Figure 2 now details $\sigma _\text {IRD}$ for all three setups. $\nu _e\bar{\nu }_e\rightarrow jj$ production, detailed in the left hand side plot, comprises only FF dipoles and, thus, only depends on $\alpha _\text { FF}$. Varying its value over four orders of magnitude leads leaves the value of $\sigma _\text {IRD}$ unchanged within the statistical uncertainties. The black line is placed at the central value of the computation with the smallest statistical uncertainty to guide the eye. As is evident, lowering the $\alpha$ parameter too much, i.e. restricting the subtraction to act only on configurations very close to the divergence, results in large cancellations between the real-subtracted and the integrated dipole contributions of Eq. (4.1), degrading the statistical prowess of the calculation. Similarly, $pp\rightarrow \nu _e\bar{nu}_e$ production, detailed on the right hand side, comprises only II dipoles and, thus, depends on $\alpha _\text { II}$ only. Showing the results for all three choices of photon content, contributing directly through the respective $\gamma q/\gamma \bar{q}$ channels as well as indirectly through the impact on the quark PDFs and, through momentum conservation, on the gluon PDF, a similar picture as for $\nu _e\bar{\nu }_e\rightarrow jj$ emerges. The three coloured lines now indicate the central value of the calculation with the smallest statistical uncertainty for each PDF choice. In each case, $\sigma _\text {IRD}$ is found to be stable under the variation of $\alpha _\text { II}$. Finally, the centre plot shows the correction contribution for the hypothetical DIS process $\nu _ep\rightarrow \nu _ej$ requiring at least one jet in the lab frame. As FI and IF dipoles always occur in pairs the $\alpha _\text { FI}$ and $\alpha _\text { IF}$ dependence is evaluated together here. The resulting $\sigma _\text {IRD}(\alpha _\text { IF}=\alpha _\text { FI})$ are also found to be stable when varying both parameters simultaneously over four orders of magnitude.

**Fig. 3:**
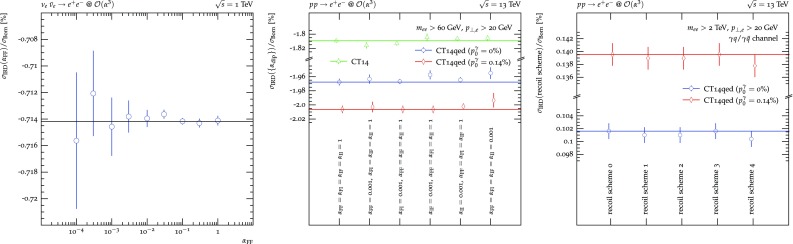
Left and centre: $\{\alpha _\text {dip}\}$-dependence of the sum of integrated subtraction term and differentially subtracted real emission for $\nu _e\bar{\nu }_e\rightarrow e^+e^-$ and $pp\rightarrow e^+e^-$. Right: dependence of the sum of the integrated subtraction term and differentially subtracted real emission for $pp\rightarrow e^+e^-$ in the $\gamma q$/$\gamma \bar{q}$ channel on the choice of recoil partner for the initial state photon splittings $c_{\tilde{k}}^\gamma$. The invariant mass of the electron pair is raised to increase the contribution of the photon induced channels. The $q\bar{q}$ and $\gamma \gamma$ channels comprise no dipoles with splitting photons

Moving away from the simplest configurations, Fig. 3 displays the results for electron-positron pair production. The left hand side plot again displays their production at the hypothetical $\nu _e$-$\bar{\nu }_e$ collider used before, finding very similar results and their independence of $\alpha _\text { FF}$. The centre plot now, however, displays the production of an electron-positron pair at the LHC. At leading order, this process proceeds through $q\bar{q}\rightarrow e^-e^+$ and $\gamma \gamma \rightarrow e^-e^+$ at $\mathcal {O}(\alpha ^2)$. Consequently, the $q\bar{q}$ channel exhibits six dipoles of all four types. In the $\gamma \gamma$ channel, the number and types of dipoles present depends on the choice of possible photon splitting spectators $c_{\tilde{k}}^\gamma$. To regulate all LO singularities the fiducial phase space is defined by requiring the dressed electrons to have a transverse momentum of at least $20\,\text {GeV}$ and the pair to have an invariant mass of at least $60\,\text {GeV}$. As $\sigma _\text {IRD}$ now potentially depends on the full set $\{\alpha _\text {dip}\}$ no continuous variation over four orders of magnitude is performed. Instead, each of the four parameters is varied independently to 0.001 keeping all others at their default value of 1. These four variations are completed by setting $\alpha _\text { FF}=\alpha _\text { FI}=\alpha _\text { IF}=\alpha _\text { II}=1$ and 0.001. The resulting correction contributions are found to be independent of $\{\alpha _\text {dip}\}$.

Finally, the right hand side plot of Fig. 3 displays the dependence of $\sigma _\text {IRD}$ on the choice of spectators in photon splittings. Only the $\gamma q/\gamma \bar{q}$ channel is considered as both the $\gamma \gamma$ and $q\bar{q}$ channels are independent of this choice in this process. The $\gamma q/\gamma \bar{q}$ channel, however, still receives contributions from photon radiation off quarks in addition to the sought after photon splittings into quark-antiquark pairs. Hence, the minimum invariant mass is raised to $2\,\text {TeV}$ to increase the relative importance of the photon PDF, enhancing the photon splitting contribution. The resulting correction contribution at $\mathcal {O}(\alpha ^3)$ is found to be independent of all five choices of photon splitting spectators available. Please note that for this process scheme 0 and 3 as well as scheme 1 and 2 lead to the same set of allowed spectators, respectively, and therefore to identical results.

**Fig. 4:**
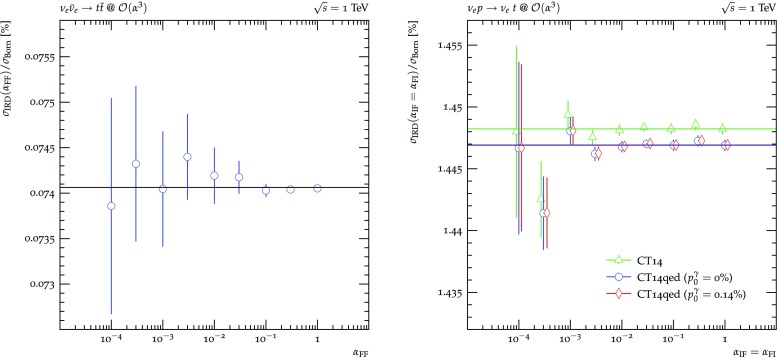
$\{\alpha _\text {dip}\}$-dependence of the sum of integrated subtraction term and differentially subtracted real emission for $\nu _e\bar{\nu }_e\rightarrow t\bar{t}$ and $\nu _e p\rightarrow \nu _e t$. For the latter the Standard Model is extended by a $u\bar{t}Z$ interaction with the structure and coupling as the existing $u\bar{u}Z$ interaction

**Massive dipoles.** Massive particles are so far only allowed in the final state.^2^ Dipoles involving massive partons, either as emitter or spectator, comprise only three types: FF, FI and IF. Emittees are always considered massless, otherwise no singularity would be present. In Fig. 4 again top-anti-top pair production at a hypothetical $\nu _e$-$\bar{\nu }_e$ collider and single top production at a hypothetical $\nu _e$-*p* collider is considered in order to study the individual dipoles separately. In the left plot, the $\alpha _\text { FF}$ (in)dependence of the $\mathcal {O}(\alpha ^3)$ correction contribution $\sigma _\text {IRD}$, containing only massive FF dipoles, is shown. It exhibits the familiar picture of decreasing statistical prowess of the calculation with too small $\alpha _\text { FF}$, but otherwise consistent results. The right plot details the $\alpha _\text { IF}$ and $\alpha _\text { FI}$ dependent massive dipoles in the hypothetical DIS scenario. As before, $\alpha _\text { IF}$ and $\alpha _\text { FI}$ are varied simultaneously for this purpose and the independence of the corrections contribution on both parameters is observed.

**Fig. 5:**
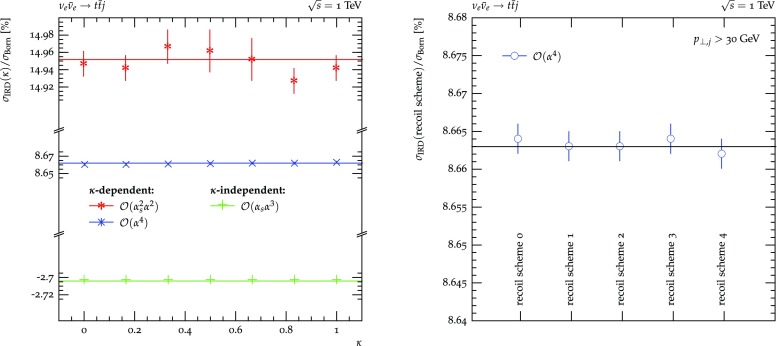
Left: $\kappa$-dependence of the sum of integrated subtraction term and differentially subtracted real emission for $\nu _e\bar{\nu }_e\rightarrow t\bar{t}j$. Right: dependence of the sum of the integrated subtraction term and differentially subtracted real emission for $\nu _e\bar{\nu }_e\rightarrow t\bar{t}j$ at $\mathcal {O}(\alpha ^4)$ on the choice of recoil partner for the final state photon splittings $c_{\tilde{k}}^\gamma$

**Fig. 6:**
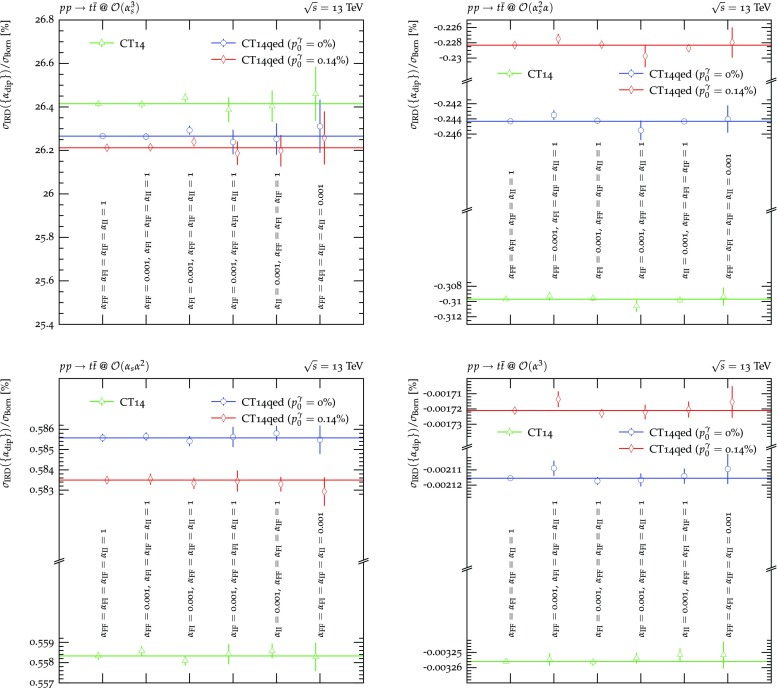
$\{\alpha _\text {dip}\}$-dependence of the sum of integrated subtraction term and differentially subtracted real emission for $\nu _e\bar{\nu }_e\rightarrow t\bar{t}$ and $pp\rightarrow t\bar{t}$

The left plot of Fig. 5 now investigates the dependence of $\sigma _\text {IRD}$ on the $\kappa$ parameter. In only arises in FF dipoles of gluons splitting into gluons or massless quarks or photons splitting into massless fermions in the presence of a massive spectator. Consequently, to restrict the number of additional contributions, top-anti-top pair production in association with one jet at the hypothetical $\nu _e$–$\bar{\nu }_e$ collider is considered. At LO, this process contributes is defined both at $\mathcal {O}(\alpha _s\alpha ^2)$ and $\mathcal {O}(\alpha ^3)$ where the final state photon forms the jet. At NLO, there are contributions at $\mathcal {O}(\alpha _s^2\alpha ^2), \mathcal {O}(\alpha _s\alpha ^3)$ and $\mathcal {O}(\alpha ^4)$. The $\mathcal {O}(\alpha _s\alpha ^3)$ contribution, however, contains neither gluon nor photon splittings. Due to the relative size of $g\rightarrow gg$ and $g\rightarrow q\bar{q}$ splittings in relation to gluon radiation off the top quarks the $\kappa$ dependence of the $\mathcal {O}(\alpha _s^2\alpha ^2)$ contribution is much more pronounced than at $\mathcal {O}(\alpha ^4)$, where photon radiation off the top quarks overwhelms the photon splitting contribution. Nonetheless, at both orders an independence of the $\sigma _\text {IRD}$ of $\kappa$ is found. In addition, the right plot investigates the influence on the choice of spectators for the final state photon splitting ocurring at $\mathcal {O}(\alpha ^4)$. No dependence on this choice is observed. Please note that for this process scheme 0 and 3 as well as scheme 1 and 2 lead to the same set of allowed spectators, respectively, and therefore to identical results.

Figure 6 now considers top-anti-top pair production at the LHC. This process occurs at LO at $\mathcal {O}(\alpha _s^2), \mathcal {O}(\alpha _s\alpha )$ and $\mathcal {O}(\alpha ^2)$. Thus, at NLO there exist four contributions, at $\mathcal {O}(\alpha _s^3)$, $\mathcal {O}(\alpha _s^2\alpha ), \mathcal {O}(\alpha _s\alpha ^2)$ and $\mathcal {O}(\alpha ^3)$. While the $\mathcal {O}(\alpha _s^3)$ and $\mathcal {O}(\alpha ^3)$ terms can be clearly identified as NLO QCD and NLO EW corrections to the LO $\mathcal {O}(\alpha _s^2)$ and $\mathcal {O}(\alpha ^2)$ expressions, respectively, the $\mathcal {O}(\alpha _s^2\alpha )$ and $\mathcal {O}(\alpha _s\alpha ^2)$ terms do not possess such a unique characterisation: they act as both NLO QCD corrections and NLO EW corrections to different LO processes. Consequently, their divergence structure contains singularities of both QCD and QED origin. Hence, both QCD and QED dipoles with underlying Born processes of different orders are needed for a full subtraction of all divergences. As explained in Sect. 3.1, this holds true both for the differential and integrated subtraction terms. It therefore serves as an additional check to verify the independence of the result of the $\{\alpha _\text {dip}\}$ for each $\mathcal {O}(\alpha _s^{3-m}\alpha ^m)$, $m=0..3$, individually. And indeed, the correction contribution $\sigma _\text {IRD}$ is found independent of $\{\alpha _\text {dip}\}$ for each such order.

**Fig. 7:**
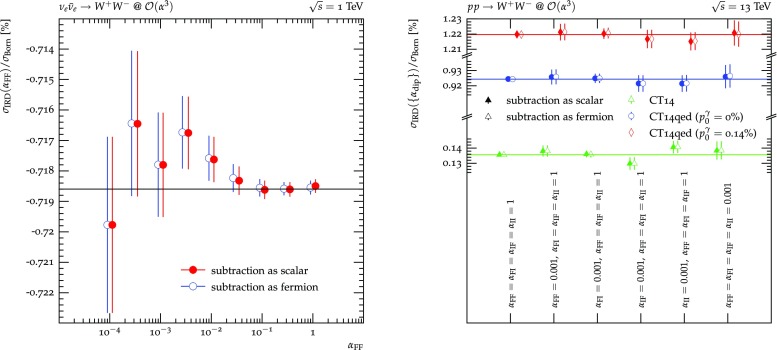
$\{\alpha _\text {dip}\}$-dependence of the sum of integrated subtraction term and differentially subtracted real emission for $\nu _e\bar{\nu }_e\rightarrow W^+W^-$ and $pp\rightarrow W^+W^-$. Both choices of subtraction terms for external massive charged vector bosons, using the ones of an external massive scalar (filled symbols) and massive fermions (empty symbols), are compared

**External** $\varvec{W}$ **bosons**. Lastly, it may become necessary to also consider external *W* bosons (or other massive charged particle with spin $>\tfrac{1}{2}$ in BSM theories) as stable final state particles, e.g. to reduce the computational complexity for high final state multiplicity processes where off-shell effects and effects in the decays can be ignored or recovered through other means [83]. In this case the literature does not provide expressions for the respective massive dipole functions. As their mass, however, can be assumed to be large enough to suppress collinear radiation well enough, this only leaves the spin-independent soft photon emission limit.^3^ Here, both the expressions for the radiation of a photon off a massive scalar or a massive fermion can be used.

Figure 7 details the production of a $W^+W^-$ pair in the hypothetical $\nu _e-\bar{\nu }_e$ collider, separating the FF dipoles and their $\alpha _\text { FF}$ dependence, on the left hand side. As before, $\sigma _\text {IRD}$ is found to be independent within the statistical accuracy and also independent of the whether the massive scalar or massive fermion subtraction terms are used. The right hand side focusses on their production at the LHC. Again, all dipoles contribute at $\mathcal {O}(\alpha ^3)$, leading again to the afore described six-point variation. Also in this case, the result is independent of $\{\alpha _\text {dip}\}$ and the choice of scalar or fermionic subtraction term. The FI and IF dipoles cannot be investigated separately, as in the $\nu _ep\rightarrow \nu _et$ case, due to charge conservation.

## Conclusions

This paper detailed the construction and implementation of the adaptation of the Catani–Seymour subtraction formalism for NLO EW calculations. Besides the translation of the QCD dipole functions to the QED case, several other issues have been addressed. They include the special role photon splittings play in the formalism, embedding extermal massive emitters of spin $>\tfrac{1}{2}$ into the formalism and the interplay of QCD and QED subtractions for processes exhibiting both kinds of divergences. The resulting general subtraction for NLO EW calculations has been implemented in the Sherpa Monte-Carlo event generator framework. Interfaces to OpenLoops, GoSam and Recola to access the needed virtual corrections exist and are fully functional.

In addition to the checks against independent implementations on the level of partial and total cross sections performed in previous publications, numerous internal cross checks for independence of technical parameter choices, $\{\alpha _\text {dip}\}=\{\alpha _\text { FF},\alpha _\text { FI},\alpha _\text { IF},\alpha _\text { II}\}$, $\kappa$ and the choice of spectator in photon splittings $c_{\tilde{k}}^\gamma$, have been presented here. This implementation will become publically available in the near future with the next major Sherpa release and an extension to the Comix matrix element generator [84] is foreseen.

## Differential splitting functions

This appendix details the complete functional form of the dipoles introduced in Eq. (2.3), $\mathcal {D}_{ij,k}, \mathcal {D}_{ij}^a, \mathcal {D}_{j,k}^{a}$ and $\mathcal {D}_j^{a,b}$. The functional forms of the spin-dependent insertions $\mathbf {V}_{ij,k}, \mathbf {V}_{ij}^a, \mathbf {V}_{j,k}^a$ and $\mathbf {V}_j^{a,b}$ and the kinematic maps of the differential splitting functions are taken from the original QCD case [40, 41]. They are summarised in the following.

### Final–final dipoles

Dipoles with both emitter and spectator in the final state take the form

A.1 $$\begin{aligned} \mathcal {D}_{ij,k}&= -\frac{1}{(p_i+p_j)^2-m_{\widetilde{\imath \jmath }}^2}\;{{\varvec{Q}}}_{{\widetilde{\imath \jmath }}{\tilde{k}}}^2{}_m\; \langle \ldots ,{\widetilde{\imath \jmath }},\ldots ,{\tilde{k}},\nonumber \\&\quad \ldots |\mathbf {V}_{ij,k}| \ldots ,{\widetilde{\imath \jmath }},\ldots ,{\tilde{k}},\ldots \rangle {}_m. \end{aligned}$$

Therein, the charge-correlator is defined in Eq. (2.5). All momenta of the dipole are on-shell

A.2 $$\begin{aligned} p_i^2&=m_i^2, \quad p_j^2=m_j^2,\quad p_{\widetilde{\imath \jmath }}^2=m_{\widetilde{\imath \jmath }}^2,\quad p_k^2=p_{\tilde{k}}^2=m_k, \end{aligned}$$

and the total four momentum flowing through it is given by

A.3 $$\begin{aligned} q&=\;p_i+p_j+p_k=p_{\widetilde{\imath \jmath }}+p_{\tilde{k}}. \end{aligned}$$

It is thus invariant under the emission. The momenta of the parton before and after the splitting are connected through the map

A.4 $$\begin{aligned} p_{\tilde{k}}&=\sqrt{\frac{\lambda \left( q^2,m_{\widetilde{\imath \jmath }}^2,m_{\tilde{k}}^2\right) }{\lambda \left( q^2,(p_i+p_j)^2,m_k^2\phantom {m_{\tilde{k}}^2}\right) }} \left( p_k-\frac{qp_k}{q^2}\,q\right) \nonumber \\&\quad +\tfrac{1}{2}\,\frac{q^2+m_{\tilde{k}}^2-m_{\widetilde{\imath \jmath }}^2}{q^2}\,q \nonumber \\ p_{\widetilde{\imath \jmath }}&= q-p_{\tilde{k}}\end{aligned}$$

wherein $m_{\tilde{k}}=m_k$ and the Kallen function is defined as $\lambda \left( a,b,c\right) =a^2+b^2+c^2-2ab-2ac-2bc$. The splitting variables read

A.5 $$\begin{aligned} y_{ij,k}&=\frac{p_ip_j}{p_ip_j+p_ip_k+p_jp_k}, \nonumber \\ z_i&=1-z_j=\frac{p_ip_k}{(p_i+p_j)p_k} \quad \text {and}\nonumber \\ z_{i,j}^{(m)}&=z_{i,j}-\frac{1-v_{ij,k}}{2}, \end{aligned}$$

which are defined on the intervals $y_{ij,k}\in [y_-,y_+]$ and $z_i\in [z_-,z_+]$. The boundaries are given by

A.6 $$\begin{aligned} \displaystyle y_-&= \frac{2m_im_j}{q_{ij,k}^2}\nonumber \\ \displaystyle z_-&= \frac{2m_i^2-q_{ij,k}^2y_{ij,k}}{2(m_i^2+m_j^2+q_{ij,k}^2y_{ij,k})}\, \left( 1-v_{ij,i}v_{ij,k}\right) \nonumber \\ \displaystyle y_+&= 1-\frac{2m_k\sqrt{q^2-m_k^2}}{q_{ij,k}^2} \nonumber \\ z_+ \displaystyle&= \frac{2m_i^2-q_{ij,k}^2y_{ij,k}}{2(m_i^2+m_j^2+q_{ij,k}^2y_{ij,k})}\, \left( 1+v_{ij,i}v_{ij,k}\right) . \end{aligned}$$

with $q_{ij,k}^2=q^2-m_i^2-m_j^2-m_k^2$. As can be seen, a divergence, residing at $y_{ij,k}=0$, is only present if either *i* or *j* are massless. The relative velocities between ${\widetilde{\imath \jmath }}$ and ${\tilde{k}}, i+j$ and *k*, and $i+j$ and *i* are given by

A.7 $$\begin{aligned} v_{{\widetilde{\imath \jmath }},{\tilde{k}}}&= \frac{\sqrt{\lambda \left( q^2,m_{\widetilde{\imath \jmath }}^2,m_{\tilde{k}}^2\right) }}{q^2-m_{\widetilde{\imath \jmath }}^2-m_{\tilde{k}}^2}\nonumber \\ v_{ij,k}&= \frac{\sqrt{\left[ 2m_k^2+q_{ij,k}^2(1-y_{ij,k})\right] ^2-4q^2m_k^2}}{q_{ij,k}^2(1-y_{ij,k})} \quad \text {and} \nonumber \\ v_{ij,i}&= \frac{\sqrt{q_{ij,k}^4y_{ij,k}^2-4m_i^2m_j^2}}{q_{ij,k}^2y_{ij,k}+2m_i^2} . \end{aligned}$$

They are introduced to facilitate the analytic integration and only take effect away from the singular limit. Thus, denoting the Spins of parton ${\widetilde{\imath \jmath }}$ in $\langle \ldots ,{\widetilde{\imath \jmath }},\ldots |\mathbf {V}_{ij,k}|\ldots ,{\widetilde{\imath \jmath }},\ldots \rangle$ by *s*, $s'$ (if ${\widetilde{\imath \jmath }}$ is a fermion), $\mu$, $\nu$ (if ${\widetilde{\imath \jmath }}$ is a photon), or omitting them (if ${\widetilde{\imath \jmath }}$ is a scalar) the dipole functions are defined as

A.8 $$\begin{aligned}&\langle s|\mathbf {V}_{\gamma f,k}|s'\rangle = 8\pi \mu ^{2\epsilon }\alpha \left\{ \frac{2}{1-z_j(1-y_{ij,k})}\right. \nonumber \\&\quad \left. -\frac{\tilde{v}_{ij,k}}{v_{ij,k}} \left[ 2-z_i(1-\epsilon )+\frac{m_f^2}{p_ip_k} \right] \right\} \delta _{ss'}\nonumber \\&\langle \mu |\mathbf {V}_{f\bar{f},k}|\nu \rangle = 8\pi \mu ^{2\epsilon }\alpha \, \frac{1}{v_{ij,k}} \nonumber \\&\quad \times \Bigg \{ -g^{\mu \nu } \left[ 1-\frac{2\kappa }{1-\epsilon }\left( z_+z_--\frac{m_f^2}{(p_i+p_j)^2}\right) \right] \nonumber \\&\quad \qquad -\frac{4}{(p_i+p_j)^2\phantom {m_f^2}} \left[ z_i^{(m)}p_i^\mu -z_j^{(m)}p_j^\mu \right] \left[ z_i^{(m)}p_i^\nu -z_j^{(m)}p_j^\nu \right] \Bigg \}\nonumber \\&\langle |\mathbf {V}_{\gamma s,k}|\rangle = 8\pi \mu ^{2\epsilon }\alpha \left\{ \frac{2}{1-z_j(1-y_{ij,k})} -\frac{\tilde{v}_{ij,k}}{v_{ij,k}} \left[ 2+\frac{m_s^2}{p_ip_k} \right] \right\} . \end{aligned}$$

It is thus clear that only in the case of photons splitting into massless fermions does the dipole splitting function have a non-diagonal structure. In all other cases, the insertion $\langle \ldots ,{\widetilde{\imath \jmath }},\ldots |\mathbf {V}_{ij,k}|\ldots ,{\widetilde{\imath \jmath }},\ldots \rangle$ reverts to a simple multiplication of a real-valued splitting function and a standard Born matrix element. The parameter $\kappa$ controls finite terms and only takes effects in the case where the respective fermionic products of the splittings are massive. Setting $\kappa =0$ somewhat simplifies the spin-dependence of the differential dipoles.

### Final–initial dipoles

Dipoles with the emitter in the final state and the spectator in the initial state take the form

A.9 $$\begin{aligned}&\mathcal {D}_{ij}^a = -\frac{1}{(p_i+p_j)^2-m_{\widetilde{\imath \jmath }}^2}\,\frac{1}{x_{ij,a}}\nonumber \\&\quad {{\varvec{Q}}}_{{\widetilde{\imath \jmath }}{\tilde{a}}}^2\; {}_m\langle \ldots ,{\widetilde{\imath \jmath }},\ldots ,{\tilde{a}},\ldots |\mathbf {V}_{ij}^a| \ldots ,{\widetilde{\imath \jmath }},\ldots ,{\tilde{a}},\ldots \rangle {}_m \end{aligned}$$

Therein, the charge-correlator is defined in Eq. (2.5). All momenta of the dipole are on-shell

A.10 $$\begin{aligned} p_i^2&=m_i^2, \quad p_j^2=m_j^2,\quad p_{\widetilde{\imath \jmath }}^2=m_{\widetilde{\imath \jmath }}^2,\quad p_a^2=p_{\tilde{a}}^2=0, \end{aligned}$$

and the total four momentum flowing through it is given by

A.11 $$\begin{aligned} q&=p_i+p_j-p_a=p_{\widetilde{\imath \jmath }}-p_{\tilde{a}}. \end{aligned}$$

It is thus invariant under the emission. The momenta of the parton before and after the splitting are connected through the map

A.12 $$\begin{aligned} p_{\tilde{a}}&= x_{ij,a}p_a \nonumber \\ p_{\widetilde{\imath \jmath }}&= q+p_{\tilde{a}}. \end{aligned}$$

The splitting variables read

A.13 $$\begin{aligned} x_{ij,a}&=1-\frac{p_ip_j-\tfrac{1}{2}\left( m_{\widetilde{\imath \jmath }}^2-m_i^2-m_j^2\right) }{(p_i+p_j)p_a} \quad \text {and}\nonumber \\ z_i&=1-z_j=\frac{p_ip_a}{(p_i+p_j)p_a}. \end{aligned}$$

The singularity of the splitting resides at $x_{ij,a}=1$ and is only present if either $m_{\widetilde{\imath \jmath }}=m_i$ or $m_{\widetilde{\imath \jmath }}=m_j$. Adopting the above convention for labeling the emitter’s spins the dipole functions are defined as

A.14 $$\begin{aligned} \langle s|\mathbf {V}_{\gamma f}^a|s'\rangle&= 8\pi \mu ^{2\epsilon }\alpha \left\{ \frac{2}{2-x_{ij,a}-z_j}\right. \nonumber \\&\quad \left. -2+z_i(1-\epsilon )-\frac{m_f^2}{p_ip_j} \right\} \delta _{ss'}\nonumber \\ \langle \mu |\mathbf {V}_{f\bar{f}}^a|\nu \rangle&= 8\pi \mu ^{2\epsilon }\alpha \left\{ -g^{\mu \nu }-\frac{4}{(p_i+p_j)^2}\right. \nonumber \\&\quad \times \left[ z_ip_i^\mu -z_jp_j^\mu \right] \left[ z_ip_i^\nu -z_jp_j^\nu \right] \Bigg \}.\nonumber \\ \langle |\mathbf {V}_{\gamma s}^a|\rangle&= 8\pi \mu ^{2\epsilon }\alpha \left\{ \frac{2}{2-x_{ij,a}-z_j}-2-\frac{m_s^2}{p_ip_j} \right\} \end{aligned}$$

Again, only photon splittings exhibit any spin correlations. In all other cases the dipole function and the underlying Born matrix element factorise.

### Initial–final dipoles

Dipoles with the emitter in the initial state and the spectator in the final state take the form

A.15 $$\begin{aligned} \mathcal {D}_{j,k}^a&= -\frac{1}{2p_ap_j}\,\frac{1}{x_{aj,k}}\;{{\varvec{Q}}}_{{\widetilde{a\;\!\!\jmath }}{\tilde{k}}}^2\; {}_m\langle \ldots ,{\widetilde{a\;\!\!\jmath }},\ldots ,{\tilde{k}},\nonumber \\&\quad \ldots |\mathbf {V}_{j,k}^a| \ldots ,{\widetilde{a\;\!\!\jmath }},\ldots ,{\tilde{k}},\ldots \rangle {}_m \end{aligned}$$

The charge-correlator is defined in Eq. (2.5). All momenta of the dipole are on-shell

A.16 $$\begin{aligned} p_a^2&= p_{\widetilde{a\;\!\!\jmath }}^2=p_i^2=0,\quad p_k^2=p_{\tilde{k}}^2=m_k^2, \end{aligned}$$

and the total four momentum flowing through it is given by

A.17 $$\begin{aligned} q&=-p_a+p_j+p_k=-p_{\widetilde{a\;\!\!\jmath }}+p_{\tilde{k}}. \end{aligned}$$

It is thus invariant under the emission. The momenta of the parton before and after the splitting are connected through the map

A.18 $$\begin{aligned} p_{\widetilde{a\;\!\!\jmath }}&= x_{ai,k}p_a\nonumber \\ p_{\tilde{k}}&= q+p_{\widetilde{a\;\!\!\jmath }}. \end{aligned}$$

The splitting variables read

A.19 $$\begin{aligned}&x_{aj,k}=1-\frac{p_jp_k}{(p_j+p_k)p_a} \quad \text {and}\quad \nonumber \\&u_j=1-u_k=\frac{p_jp_a}{(p_j+p_k)p_a}. \end{aligned}$$

As the splitting partons are all in the initial state and therefore massless, the singularity at $u_j=0$ is present in any case. Adopting the above convention for labeling the spin of the emitter ${\widetilde{a\;\!\!\jmath }}$ the dipole functions are defined as

A.20 $$\begin{aligned} \langle s|\mathbf {V}_{\gamma ,k}^f|s'\rangle&= 8\pi \mu ^{2\epsilon }\alpha \left\{ \frac{2}{2-x_{aj,k}-u_k}-1-x_{aj,k} \right. \nonumber \\&\left. \quad -\epsilon (1-x_{aj,k}) \right\} \delta _{ss'}\nonumber \\ \langle s|\mathbf {V}_{f,k}^\gamma |s'\rangle&= 8\pi \mu ^{2\epsilon }\alpha \left\{ 1-\epsilon -2x_{aj,k}(1-x_{aj,k})\phantom {\frac{2}{x_{aj,k}}} \right\} \delta _{ss'}\nonumber \\ \langle \mu |\mathbf {V}_{f,k}^f|\nu \rangle&= 8\pi \mu ^{2\epsilon }\alpha \left\{ -g^{\mu \nu }x_{aj,k} -\frac{1-x_{aj,k}}{x_{aj,k}}\frac{2z_jz_k}{p_jp_k}\right. \nonumber \\&\quad \times \left. \left[ \frac{p_j^\mu }{u_j}-\frac{p_k^\mu }{u_k}\right] \left[ \frac{p_j^\nu }{u_j}-\frac{p_k^\nu }{u_k}\right] \right\} \end{aligned}$$

As only massless particles are considered as initial states, no splitting functions involving massive scalars are needed.

### Initial–initial dipoles

Dipoles with both emitter and spectator in the initinal state take the form

A.21 $$\begin{aligned} \mathcal {D}_j^{a,b}&= -\frac{1}{2p_ap_j}\,\frac{1}{x_{aj,b}}\;{{\varvec{Q}}}_{{\widetilde{a\;\!\!\jmath }}{\tilde{b}}}^2\; {}_m\langle \ldots ,{\widetilde{a\;\!\!\jmath }},\ldots ,{\tilde{b}},\nonumber \\&\quad \ldots |\mathbf {V}_j^{a,b}| \ldots ,{\widetilde{a\;\!\!\jmath }},\ldots ,{\tilde{b}},\ldots \rangle {}_m \end{aligned}$$

The charge-correlator is defined in Eq. (2.5). All momenta of the dipole are on-shell

A.22 $$\begin{aligned} p_a^2&=p_{\widetilde{a\;\!\!\jmath }}^2=p_i^2=0,\quad p_b^2=p_{\tilde{b}}^2=0, \end{aligned}$$

and the total four momentum flowing through it is given by

A.23 $$\begin{aligned} q&=-p_a-p_b+p_j,\quad {\tilde{q}}=-p_{\widetilde{a\;\!\!\jmath }}-p_{\tilde{b}}. \end{aligned}$$

before and after the emission, respectively. Although *q* acquires transverse momentum after the emission, $q^2={\tilde{q}}^2$ holds, i.e. the mass of the dipole is invariant. The momenta of the partos before and after the splitting are connected through the map

A.24 $$\begin{aligned}&p_{\widetilde{a\;\!\!\jmath }}= x_{ai,b}p_a \nonumber \\&p_{\tilde{b}}= p_b. \end{aligned}$$

While the initial state cannot absorb recoil transverse to the beam access, it is transferred to the final state through a collective boost

A.25 $$\begin{aligned} k_{\tilde{\imath }}^\mu&= k_i^\nu \left[ \delta _\nu ^\mu -2\,\frac{(q+{\tilde{q}})_\nu (q+{\tilde{q}})^\mu }{(q+{\tilde{q}})^2} +2\,\frac{q_\nu {\tilde{q}}^\mu }{q^2} \right] \end{aligned}$$

of all final state partons. The splitting variables read

A.26 $$\begin{aligned} x_{aj,b}=1-\frac{p_j(p_a+p_b)}{p_ap_b} \quad \text {and}\quad v_j=\frac{p_ap_j}{p_ap_b}, \quad v_b=1. \end{aligned}$$

As the splitting partons are all in the initial state and therefore massless, the singularity at $v_j=0$ is present in any case. Adopting the above convention for labeling the spin of the emitter ${\widetilde{a\;\!\!\jmath }}$ the dipole functions are defined as

A.27 $$\begin{aligned} \langle s|\mathbf {V}_\gamma ^{f,b}|s'\rangle&= 8\pi \mu ^{2\epsilon }\alpha \left\{ \frac{2}{2-x_{aj,b}}-1-x_{aj,b}\right. \nonumber \\&\quad \left. -\,\epsilon (1-x_{aj,b}) \right\} \delta _{ss'}\nonumber \\ \langle s|\mathbf {V}_f^{\gamma ,b}|s'\rangle&= 8\pi \mu ^{2\epsilon }\alpha \left\{ 1-\epsilon -2x_{aj,b}(1-x_{aj,b})\phantom {\frac{2}{x_{aj,b}}} \right\} \delta _{ss'}\nonumber \\ \langle \mu |\mathbf {V}_f^{f,b}|\nu \rangle&= 8\pi \mu ^{2\epsilon }\alpha \left\{ -g^{\mu \nu }x_{aj,b} -\frac{1-x_{aj,b}}{x_{aj,b}}\frac{2v_jv_b}{p_jp_b}\right. \nonumber \\&\quad \times \left. \left[ \frac{p_j^\mu }{v_j}-\frac{p_b^\mu }{v_b}\right] \left[ \frac{p_j^\nu }{v_j}-\frac{p_b^\nu }{v_b}\right] \right\} \end{aligned}$$

As only massless particles are considered as initial states, no splitting functions involving massive scalars are needed.

## Integrated splitting functions

This appendix summarises the complete functional form of the integrated dipole terms cast in the form of the ${{\varvec{I}}}$, ${{\varvec{K}}}$ and ${{\varvec{P}}}$ operators [40, 41].

### Terms of the ${{\varvec{I}}}\,$perator

Each dipole contribution to the ${{\varvec{I}}}$ operator reads

B.1 $$\begin{aligned} {{\varvec{I}}}_{ik}(\epsilon ,\mu ^2;\kappa ,\{\alpha _\text {dip}\})&= {{\varvec{Q}}}_{ik}^2 \left[ \mathcal {V}_{ik}(\epsilon ,\mu ^2;\kappa ) +\Gamma _i(\epsilon ,\mu ^2)\right. \nonumber \\&\quad \left. +\,\gamma _i\left( 1+\ln \frac{\mu ^2}{s_{ik}}\right) +K_i \right. \nonumber \\&\quad \left. +\,A_{ik}^I(\{\alpha _\text {dip}\}) +\mathcal {O}(\epsilon ) \right] , \end{aligned}$$

cf. Eq. (2.8). Therein, $\mathcal {V}_{ik}$ takes the general form

B.2 $$\begin{aligned}&\mathcal {V}_{ik}(\epsilon ,\mu ^2;\kappa ) \nonumber \\&\quad =\,\left\{ \begin{array}{ll} Q_i^2 \left( \frac{\mu ^2}{s_{ik}}\right) ^\epsilon \left[ \mathcal {V}^\text {(S)}_{ik}(\epsilon )+\mathcal {V}^\text {(NS)}_{ik}(\kappa )-\tfrac{\pi ^2}{3}\right] &{} i\ne \gamma \\ \phantom {\int \limits _A^B}\mathcal {V}^\text {(NS)}_{\gamma k}(\kappa ) &{} i=\gamma . \end{array}\right. \end{aligned}$$

Wherein, $\mathcal {V}^\text {(S)}_{ik}$ contains all infrared singularities (double poles for massless fermion splittings, single pole for massive splittings), and a non-singular piece $\mathcal {V}^\text {(NS)}_{ik}$ that incorporates all finite dependences on the masses of the emitter and the spectator. The latter vanishes in the massless case, see below. In the case of photon splittings, only the non-singular part survives, again being non-zero only in the case of massive spectators. The singular part vanishes in case of photon splittings.

The singular term $\mathcal {V}^\text {(S)}_{ik}$ is symmetric in *i* and *k*, it is given by

B.3 $$\begin{aligned} \mathcal {V}^\text {(S)}_{ik}(\epsilon )&=\frac{1}{v_{ik}}\left( \frac{q_{ik}^2}{s_{ik}}\right) ^\epsilon \left[ \frac{1}{\epsilon ^2} \left( 1-\tfrac{1}{2}\rho _i^{-2\epsilon }-\tfrac{1}{2}\rho _k^{-2\epsilon } \right) \right. \nonumber \\&\qquad \qquad \qquad \left. -\frac{\pi ^2}{12}\left( \Theta (m_i)+\Theta (m_k)\right) \right] \end{aligned}$$

with

B.4 $$\begin{aligned} \rho _{i/k}&=\sqrt{\frac{(1-v_{ik})s_{ik}+2m_{i/k}^2}{(1+v_{ik})s_{ik}+2m_{i/k}^2}} {\mathop {\longrightarrow }\limits ^{m_{i/k}\rightarrow 0}} 0 \end{aligned}$$

and the velocity factors

B.5 $$\begin{aligned} v_{ik}&=\frac{\sqrt{\lambda \left( q_{ik}^2,m_i^2,m_k^2\right) }}{s_{ik}} {\mathop {\longrightarrow }\limits ^{m_i,m_k\rightarrow 0}} 1 \end{aligned}$$

parametrise the mass dependence. Therein, $q_{ik}^2=(p_i+p_k)^2=s_{ik}+m_i^2+m_k^2$ is the dipole invariant mass. In $d=4-2\epsilon$ dimensions, $\epsilon >0$, the massless limit is approached smoothly. Subsequently taking the limit $\epsilon \rightarrow 0$, however, results in $\epsilon ^{-2}$ poles only if at least one of both particles making up the dipole is massless. Completely massive dipoles only result in single infrared poles.

The non-singular $\mathcal {V}^\text {(NS)}_{ik}$ contributions encode the additional finite $m_{i/k}>0$ contributions. They vanish in the limit that both $m_i\rightarrow 0$ and $m_k\rightarrow 0$ and their precise form can be found in Eq. (6.21)–(6.26) of [41]. Of particular interest is the term for a photon emitter in the presence of a massive spectator,

B.6 $$\begin{aligned} \mathcal {V}^\text {(NS)}_{\gamma k}(\kappa )&= \gamma _\gamma \left[ \log \frac{s_{ik}}{q_{ik}^2} -2\log \frac{|q_{ik}|-m_k}{|q_{ik}|} -\frac{2m_k}{|q_{ik}|+m_k} \right] \nonumber \\&\quad +\frac{\pi ^2}{6}-\text {Li}_2\left( \frac{s_{ik}}{q_{ik}^2}\right) \nonumber \\&\quad +2\gamma _\gamma \left( \kappa -\tfrac{2}{3}\right) \frac{m_k^2}{s_{ik}} \log \frac{2m_k}{|q_{ik}|+m_k}, \end{aligned}$$

with $|q_{ik}|=\sqrt{q_{ik}^2}$. Thus, choosing $\kappa =\tfrac{2}{3}$ somewhat simplifies the integrated subtraction term.

Further,

B.7 $$\begin{aligned} \begin{aligned} \Gamma _i(\epsilon ,\mu ^2) \,=\; \left\{ \begin{array}{ll} \phantom {\int \limits _A^B}\frac{1}{\epsilon }\,\gamma _i &{} i=\text {massless fermion} \\ Q_i^2\left[ \frac{1}{\epsilon }+\tfrac{1}{2}\ln \frac{m_i^2}{\mu ^2}-2\right] &{} i=\text {massive fermion} \\ \phantom {\int \limits _A^B}\frac{1}{\epsilon }\,\gamma _\gamma &{} i=\gamma \\ Q_i^2\left[ \frac{1}{\epsilon }+\ln \frac{m_i^2}{\mu ^2}-2\right] &{} i=\text {massive scalar}. \end{array}\right. \end{aligned} \end{aligned}$$

In case of the photon, the sum runs over all massless charged flavours present in the model. The flavour constants $\gamma _i$ and $K_i$ are given by

B.8 $$\begin{aligned} \gamma _i&= \left\{ \begin{array}{ll} \tfrac{3}{2}Q_i^2 &{} i=\text {fermion} \\ \phantom {\int \limits _A^B}- \tfrac{2}{3}\sum \limits _f N_{C,f}\,Q_f^2 &{} i=\gamma \\ 2Q_i^2 &{} i=\text {scalar} \end{array}\right. \nonumber \\ K_i&= \left\{ \begin{array}{ll} \left( \tfrac{7}{2}-\tfrac{\pi ^2}{6}\right) Q_i^2 &{} i=\text {fermion} \\ \phantom {\int \limits _A^B}-\tfrac{10}{9}\sum \limits _f N_{C,f}\,Q_f^2 &{} i=\gamma \\ \left( 4-\tfrac{\pi ^2}{6}\right) Q_i^2 &{} i=\text {scalar} \end{array}\right. \end{aligned}$$

and are independent of the particle masses. For the photon, the sums run over all charged massless flavours of the model, $N_{C,f}$ being their respective colour multiplicity. Lastly, the dependence of the ${{\varvec{I}}}$ operator on the technical parameters $\{\alpha _\text {dip}\}=\{\alpha _\text { II},\alpha _\text { IF},\alpha _\text { FI},\alpha _\text { FF}\}$ is encoded in the last term $A_{ik}^I(\{\alpha _\text {dip}\})$ which is detailed in App. C.

### Terms of the ${{\varvec{K}}}\,$and ${{\varvec{P}}}\,$operators

The ${{\varvec{K}}}$ operator of Eq. (2.9) reads

B.9

Therein, the $\overline{K}$ collect universal terms present in all splitting involving an initial state as either emitter or spectator. They are defined as

B.10 $$\begin{aligned} \overline{K}_{aa'}(x)&= P_\text {reg}^{aa'}(x)\log \frac{1-x}{x}+\hat{P}^{\prime aa'}(x)\nonumber \\&\quad +\delta ^{aa'} \left[ Q_a^2\left( \frac{2}{1-x}\,\log \frac{1-x}{x}\right) _+\right. \nonumber \\&\qquad \quad \left. -\delta (1-x)\left( \gamma _a+K_a-\frac{5}{6}\,\pi ^2Q_a^2\right) \right] . \end{aligned}$$

As can be seen, they are independent of the final state and thus independent of any final state particle masses. Here, the regular part of the Altarelli–Parisi splitting functions are given by

B.11 $$\begin{aligned} P_\text {reg}^{aa'}(x)&= \left\{ \begin{array}{ll} -Q_a^2\, (1+x) &{} aa'=ff\\ Q_a^2\, [x^2+(1-x)^2] &{} aa'=f\gamma \\ N_{C,a'}Q_{a'}^2\,\frac{1+(1-x)^2}{x} &{} aa'=\gamma f\\ 0 &{} aa'=\gamma \gamma , \end{array} \right. \end{aligned}$$

with $N_{C,a'}$ the multiplicity in the QCD representation of parton $a'$ (3 for quarks, 1 for leptons). Similarly, the $\epsilon$-dependent part of the splitting functions is defined as

B.12 $$\begin{aligned} \hat{P}^{\prime aa'}(x)&= \left\{ \begin{array}{ll} Q_a^2\, (1-x) &{} aa'=ff\\ Q_a^2\, x &{} aa'=f\gamma \\ N_{C,a'}Q_{a'}^2\,x(1-x) &{} aa'=\gamma f\\ 0 &{} aa'=\gamma \gamma . \end{array} \right. \end{aligned}$$

Thus, for $a=a'=\gamma$ the $\overline{K}$ term takes the simple form

B.13 $$\begin{aligned} \overline{K}_{\gamma \gamma }(x)&= -\tfrac{8}{3}\,\gamma _\gamma \,\delta (1-x), \end{aligned}$$

and comprises an end-point term only. The factorisation scheme dependent terms $K_\text {FS}$ vanish in the $\overline{\text {MS}}$ scheme, and the $\mathcal {K}_{i,aa'}$, containing remnants from final state splittings where the initial state *a* forms the spectator. As it also depends on the final state flavour *i*, its forms are given as follows. The contributions, listed separately for scalars, fermions and photons, read

B.14 $$\begin{aligned} \mathcal {K}_{f,\gamma f}(x)&= 0\nonumber \\ \mathcal {K}_{f,ff}(x)&= 2 \left[ \left( \frac{\log (1-x)}{1-x}\right) _+ -\frac{\log (2-x)}{1-x} \right] \nonumber \\&\quad +\left[ J_{\gamma Q}^a\left( x,\frac{m_i}{\sqrt{s_{ik}}}\right) \right] _+ \nonumber \\&\quad +2\left( \frac{1}{1-x}\right) _+ \log \frac{(2-x)s_{ik}}{(2-x)s_{ik}+m_i^2} \nonumber \\&\quad +\delta (1-x)\left[ -\frac{\gamma _f}{Q_i^2} +\frac{m_i^2}{s_{ik}}\,\log \frac{m_i^2}{s_{ik}+m_i^2} +\tfrac{1}{2}\frac{m_i^2}{s_{ik}+m_i^2} \right] \nonumber \\ \mathcal {K}_{f,f\gamma }(x)&= 2\,\frac{m_i^2}{xs_{ik}}\,\log \frac{m_i^2}{(1-x)s_{ik}+m_i^2}\nonumber \\ \mathcal {K}_{f,\gamma \gamma }(x)&= \mathcal {K}_{f,ff}, \end{aligned}$$

and

B.15 $$\begin{aligned} \mathcal {K}_{s,aa'}(x)&= \mathcal {K}_{f,aa'}(x) -\delta ^{aa'} \left[ \left( \frac{s_{ik}^2(1-x)}{2[s_{ik}(1-x)+m_i^2]^2}\right) _+\right. \nonumber \\&\quad \left. -\delta (1-x) \left( \frac{m_i^2}{s_{ik}}\log \frac{m_i^2}{q_{ik}^2}+\frac{m_i^2}{2q_{ik}^2} +\frac{\gamma _s-\gamma _f}{Q_s^2} \right) \right] . \end{aligned}$$

The missing function $J_{\gamma Q}^a(x,y)$ is given in Eq. (5.58) of [41]. In the massless limit this reduces to

B.16 $$\begin{aligned} \mathcal {K}_{i,aa'}(x)&= -\delta ^{aa'} \frac{\gamma _i}{Q_i^2} \left[ \left( \frac{1}{1-x}\right) _++\delta (1-x)\right] \end{aligned}$$

Simultaneously, for $$i=\gamma $$

B.17 $$\begin{aligned} \mathcal {K}_{\gamma ,aa'}(x)&= -\delta ^{aa'}\gamma _\gamma \left[ \left( \frac{1}{1-x}\right) _++\delta (1-x)\right] \end{aligned}$$

due to the different normalisation of ${{\varvec{Q}}}_{ik}^2$ in the photon case. The $K_{aa',k}^t$ terms comprise of initial state splittings in the presence of a final state spectator. They take the form

B.18 $$\begin{aligned} K_{aa',k}^t(x)&= P_\text {reg}^{aa'}(x)\,\log \frac{(1-x)s_{ak}}{(1-x)s_{ak}+m_k^2}+\gamma _a\,\delta ^{aa'}\,\delta (1-x)\nonumber \\&\quad \times \left[ \log \frac{s_{ak}-2m_k\sqrt{s_{ak}+m_k^2} +2m_k^2}{s_{ak}}\right. \nonumber \\&\quad \quad \qquad \left. +\frac{2m_k}{\sqrt{s_{ak}+m_k^2}+m_k} \right] \end{aligned}$$

Again, for $a=a'=\gamma$ the end-point contribution cannot arise as no corresponding splitting is integrated over, $K_{\gamma \gamma ,k}^t=0$. Finally, the $\tilde{K}$ terms, containing correlations between both initial state partons, read

B.19 $$\begin{aligned} \tilde{K}_{aa'}(x)&= P_\text {reg}^{aa'}(x)\,\log (1-x)\nonumber \\&\quad +Q_{a'}^2\delta ^{aa'}\left[ 2\left( \frac{\log (1-x)}{1-x}\right) _+-\frac{\pi ^2}{3}\,\delta (1-x)\right] . \end{aligned}$$

They thus vanish for $a=a'=\gamma$.

Finally, the ${{\varvec{P}}}$ operator is given by

B.20 $$\begin{aligned}&{{\varvec{P}}}_{aa'}(x,\mu _F^2)\nonumber \\&\quad = \frac{\alpha }{2\pi }\;P^{aa'}(x) \left[ \sum \limits _k{{\varvec{Q}}}_{a'k}^2\,\log \frac{\mu _\text {F}^2}{xs_{ak}} +{{\varvec{Q}}}_{a'b}^2\,\log \frac{\mu _\text {F}^2}{xs_{ab}} \right] , \end{aligned}$$

cf. Eq. (2.10). Therein, the regularised Altarelli-Parisi splitting functions are given by

B.21 $$\begin{aligned} P^{aa'}(x)&= P_\text {reg}^{aa'}(x) +\delta ^{aa'} \left[ 2\,Q_a^2\left( \frac{1}{1-x}\right) _++\gamma _a\,\delta (1-x) \right] . \end{aligned}$$

Most importantly,

B.22 $$\begin{aligned} P^{\gamma \gamma }(x)&= \gamma _\gamma \,\delta (1-x). \end{aligned}$$

## Precise forms of $A_{ik}^I$ and $A_{ab}^K$

### The $A_{ik}^I$ term

The explicit dependence of the ${{\varvec{I}}}$ operator on the $\{\alpha _\text {dip}\}$ parameters, or more precisely only on $\alpha _\text { FF}$, is given by [54, 55, 86–88] if *i* is a massless fermion

C.1 $$\begin{aligned} A_{ik}^I(\{\alpha \})&= -\ln ^2\alpha _\text { FF}-\gamma _i\left( \ln \alpha _\text { FF}+1-\alpha _\text { FF}\right) \quad m_k=0 \nonumber \\&= \ln ^2\frac{1-y_+^2+2xy_+}{(1-x+y_+)(1+x-y_+)} -2\ln ^2\frac{1+y_+-x}{1+y_+} \nonumber \\&\quad +\,4\left[ \ln \frac{1+y_+}{2}\ln \frac{1-y_++x}{1-y_+}\right. \nonumber \\&\qquad \quad \left. +\,\ln \frac{1+y_+}{2y_+}\ln \frac{1-y_+^2+2x_+y_+}{1-y_+^2} \right. \nonumber \\&\qquad \quad \left. +\,\text {Li}_2\left( \frac{1-y_+}{1+y_+}\right) -\text {Li}_2\left( \frac{1-y_+}{2}\right) \right. \nonumber \\&\qquad \quad \left. +\,\text {Li}_2\left( \frac{1+x-y_+}{2}\right) -\text {Li}_2\left( \frac{1-y_+^2+2xy_+}{(1+y_+)^2}\right) \right] \nonumber \\&\quad -\tfrac{3}{2}\left( \ln \alpha _\text { FF}+y_+(1-\alpha _\text { FF})\right) \quad m_k\ne 0 \end{aligned}$$

or if *i* a massive fermion

C.2 $$\begin{aligned} A_{ik}^I(\{\alpha \})&= 2\left[ -\ln \alpha _\text { FF}\left( 1+\ln \mu _i^2\right) +\text {Li}_2\left( \alpha _\text { FF}\frac{\mu _i^2-1}{\mu _i^2}\right) \right. \nonumber \\&\quad \qquad \left. -\text {Li}_2\left( \frac{\mu _i^2-1}{\mu _i^2}\right) \right] \nonumber \\&\quad +\tfrac{1}{2}\left[ -(1-\alpha _\text { FF})\frac{3\alpha _\text { FF}(1-\mu _i^2)+2\mu _i^2}{\alpha _\text { FF}+(1-\alpha _\text { FF})\mu _i^2} \right. \nonumber \\&\quad \qquad \left. +\frac{1+\mu _i^2}{1-\mu _i^2} \ln \left( \alpha _\text { FF}+(1-\alpha _\text { FF})\mu _i^2\right) \right] \nonumber \\&\quad m_k=0 \nonumber \\&= \tfrac{3}{2}(1+\alpha _\text { FF}y_+) +\frac{1}{1-\mu _k} -\frac{2-2\mu _i^2-\mu _k}{d} \nonumber \\&\quad +\tfrac{1}{2}\frac{(1-\alpha _\text { FF}y_+)\mu _i^2}{\mu _i^2+2\alpha _\text { FF}y_+d} -2\ln \alpha _\text { FF}\nonumber \\&\quad +\tfrac{1}{2}\frac{\mu _i^2+d}{d} \ln \frac{\mu _i^2+2\alpha _\text { FF}y_+d}{(1-\mu _k)} \nonumber \\&\quad +\frac{2}{v_{ik}}\left[ {} -\text {Li}_2\left( \frac{a+x}{a+x_+}\right) +\text {Li}_2\left( \frac{a}{a+x_+}\right) \right. \nonumber \\&\quad \qquad \left. +\,\text {Li}_2\left( \frac{x_--x}{x_-+a}\right) -\text {Li}_2\left( \frac{x_-}{x_-+a}\right) \right. \nonumber \\&\quad \qquad \left. +\,\text {Li}_2\left( \frac{c+x}{c+x_+}\right) -\text {Li}_2\left( \frac{c}{c+x_+}\right) \right. \nonumber \\&\quad \qquad \left. -\,\text {Li}_2\left( \frac{x_--x}{x_-+c}\right) +\text {Li}_2\left( \frac{x_-}{x_-+c}\right) \right. \nonumber \\&\quad \qquad \left. -\,\text {Li}_2\left( \frac{b-x}{b-x_-}\right) +\text {Li}_2\left( \frac{b}{b-x_-}\right) \right. \nonumber \\&\quad \qquad \left. +\,\text {Li}_2\left( \frac{x_+-x}{x_+-b}\right) -\text {Li}_2\left( \frac{x_+}{x_+-b}\right) \right. \nonumber \\&\quad \qquad \left. +\,\text {Li}_2\left( \frac{b-x}{b+a}\right) -\text {Li}_2\left( \frac{b}{b+a}\right) \right. \nonumber \\&\quad \qquad \left. -\,\text {Li}_2\left( \frac{c+x}{c-a}\right) +\text {Li}_2\left( \frac{c}{c-a}\right) \right. \nonumber \\&\quad \qquad \left. +\ln (c+x) \ln \frac{(a-c)(x_+-x)}{(a+x)(x_++c)}\right. \nonumber \\&\quad \qquad \left. -\ln c \ln \frac{(a-c)x_+}{a(x_++c)} \right. \nonumber \\&\quad \qquad \left. -\ln (b-x) \ln \frac{(a+b)(x_--x)}{(a+x)(x_--b)} \right. \nonumber \\&\quad \qquad \left. +\ln b \ln \frac{(a+b)x_-}{a(x_--b)} \right. \nonumber \\&\quad \qquad \left. -\ln \left( (a+x)(b-x_+)\right) \ln (x_+-x)\right. \nonumber \\&\quad \qquad \left. +\ln \left( a(b-x_+)\right) \ln x_+ \right. \nonumber \\&\quad \qquad \left. +\ln d \ln \frac{(a+x)x_+x_-}{a(x_+-x)(x_--x)} \right. \nonumber \\&\quad \qquad \left. +\ln \frac{x_--x}{x_-} \ln \frac{c+x_-}{a+x_-} \right. \nonumber \\&\quad \qquad \left. +\tfrac{1}{2}\ln \frac{a+x}{a}\ln \left( a(a+x)(a+x_+)^2\right) \right] \nonumber \\&\quad m_k\ne 0 \end{aligned}$$

or if *i* is a photon

C.3 $$\begin{aligned}&= -\gamma _i\left( \ln \alpha _\text { FF}+1-\alpha _\text { FF}\right) \quad m_k=0 \nonumber \\&= -\gamma _i \left[ \left( \frac{1-\mu _k-\alpha _\text { FF}y_+(1+\mu _k)}{1+\mu _k} +\ln \frac{\alpha _\text { FF}y_+(1+\mu _k)}{1-\mu _k}\right) \right. \nonumber \\&\quad \qquad \left. +\tfrac{3}{2}(\kappa -\tfrac{2}{3})\frac{2\mu _k^2}{1-\mu _k^2} \ln \frac{(1-\alpha _\text { FF}y_+)(1+\mu _k)}{2\mu _k} \right] \nonumber \\&\quad m_k\ne 0 \end{aligned}$$

or a massive scalar

C.4 $$\begin{aligned}&= 2\left[ -\ln \alpha _\text { FF}(1+\ln \mu _i^2) +\text {Li}_2\left( \alpha _\text { FF}\frac{\mu _i^2-1}{\mu _i^2}\right) \right. \nonumber \\&\quad \left. -\text {Li}_2\left( \frac{\mu _i^2-1}{\mu _i^2}\right) -(1-\alpha _\text { FF}) \frac{\alpha _\text { FF}(1-\mu _i^2)+\mu _i^2}{\alpha _\text { FF}+(1-\alpha _\text { FF})\mu _i^2} \right] \nonumber \\&\quad m_k=0 \nonumber \\&= \tfrac{3}{2}(1+\alpha _\text { FF}y_+) +\frac{1}{1-\mu _k} -\frac{2-2\mu _i^2-\mu _k}{d} \nonumber \\&\quad +\tfrac{1}{2}\frac{(1-\alpha _\text { FF}y_+)\mu _i^2}{\mu _i^2+\alpha _\text { FF}y_+ d} -2\ln \alpha _\text { FF}\nonumber \\&\quad \qquad -\tfrac{1}{2}(1-2\alpha _\text { FF})y_+ \frac{\alpha _\text { FF}y_+ d (1-\mu _k)+\mu _i^2}{(2\alpha _\text { FF}y_+ d+\mu _i^2)(1-\mu _k)} \nonumber \\&\quad \qquad +\frac{2}{v_{ik}}\left[ {} -\text {Li}_2\left( \frac{a+x}{a+x_+}\right) +\text {Li}_2\left( \frac{a}{a+x_+}\right) \right. \nonumber \\&\quad \qquad \left. +\text {Li}_2\left( \frac{x_--x}{x_-+a}\right) -\text {Li}_2\left( \frac{x_-}{x_-+a}\right) \right. \nonumber \\&\quad \qquad \left. +\,\text {Li}_2\left( \frac{c+x}{c+x_+}\right) -\text {Li}_2\left( \frac{c}{c+x_+}\right) \right. \nonumber \\&\quad \qquad \left. -\,\text {Li}_2\left( \frac{x_--x}{x_-+c}\right) +\text {Li}_2\left( \frac{x_-}{x_-+c}\right) \right. \nonumber \\&\quad \qquad \left. -\,\text {Li}_2\left( \frac{b-x}{b-x_-}\right) +\text {Li}_2\left( \frac{b}{b-x_-}\right) \right. \nonumber \\&\quad \qquad \left. +\,\text {Li}_2\left( \frac{x_+-x}{x_+-b}\right) -\text {Li}_2\left( \frac{x_+}{x_+-b}\right) \right. \nonumber \\&\quad \qquad \left. +\,\text {Li}_2\left( \frac{b-x}{b+a}\right) -\text {Li}_2\left( \frac{b}{b+a}\right) \right. \nonumber \\&\quad \qquad \left. -\,\text {Li}_2\left( \frac{c+x}{c-a}\right) +\text {Li}_2\left( \frac{c}{c-a}\right) \right. \nonumber \\&\quad \qquad \left. +\ln (c+x) \ln \frac{(a-c)(x_+-x)}{(a+x)(x_++c)} \right. \nonumber \\&\quad \qquad \left. -\ln c \ln \frac{(a-c)x_+}{a(x_++c)} \right. \nonumber \\&\quad \qquad \left. -\ln (b-x) \ln \frac{(a+b)(x_--x)}{(a+x)(x_--b)} \right. \nonumber \\&\quad \qquad \left. +\ln b \ln \frac{(a+b)x_-}{a(x_--b)} \right. \nonumber \\&\quad \qquad \left. -\ln \left( (a+x)(b-x_+)\right) \ln (x_+-x)\right. \nonumber \\&\quad \qquad \left. +\ln \left( a(b-x_+)\right) \ln x_+ \right. \nonumber \\&\quad \qquad \left. +\ln d \ln \frac{(a+x)x_+x_-}{a(x_+-x)(x_--x)}\right. \nonumber \\&\quad \qquad \left. +\ln \frac{x_--x}{x_-} \ln \frac{c+x_-}{a+x_-} \right. \nonumber \\&\quad \qquad \left. +\tfrac{1}{2}\ln \frac{a+x}{a}\ln \left( a(a+x)(a+x_+)^2\right) \right] \nonumber \\&\quad m_k\ne 0 \end{aligned}$$

As can be seen, the massless case can be recovered from the massive one in the limit that $m_k\rightarrow 0$. The $A_{ik}^I$ term could in principle also be shifted into the ${{\varvec{K}}}$ operator to leave the ${{\varvec{I}}}$ operator invariant. To be applicable to pure final-state calculations, though, it is left where it is. The various abbreviations above are defined as

C.5 $$\begin{aligned}&\mu _{i/k}^2 =\frac{m_{i/k}^2}{q_{ik}^2}\nonumber \\&y_+=\frac{(1-\mu _k)^2-\mu _i^2}{1-\mu _i^2-\mu _k^2} \,=\; \left\{ \begin{array}{ll} 1 &{} \text {if } m_k=0 \\ \frac{1-\mu _k}{1+\mu _k} &{} \text {if } m_i=0 \end{array} \right. \nonumber \\&y=\tfrac{1}{2}\Bigg [1-\mu _k^2+\alpha _\text { FF}(1-\mu _k)^2 -(1-\mu _k)\nonumber \\&\quad \times \left. \sqrt{(\alpha _\text { FF}(1-\mu _k))^2+(1+\mu _k)^2 -2\alpha _\text { FF}(1+\mu _k^2)}\right] \nonumber \\&x=(1-\alpha _\text { FF})y_+ +\sqrt{(1-\alpha _\text { FF}) \left( 1 - \alpha _\text { FF}y_+^2 -\frac{4\mu _i^2\mu _k^2}{d^2}\right) }\nonumber \\&x_\pm =\tfrac{1}{4}y_+\pm \tfrac{1}{2}\frac{\sqrt{\lambda \left( 1,\mu _i^2,\mu _k^2\right) }}{d}\nonumber \\&a=\frac{\mu _k}{d}\nonumber \\&b=\frac{1-\mu _k}{d}\nonumber \\&c=abd\,=\;1-y_+\nonumber \\&d=\tfrac{1}{2}\frac{s_{ik}}{q_{ik}^2}\,=\;\tfrac{1}{2}(1-\mu _i^2-\mu _k^2) \end{aligned}$$

### The $A_{ab}^K$ term

The dependence of the ${{\varvec{K}}}$ operator on the $\{\alpha _\text {dip}\}$ parameters, or more precisely only on $\alpha _\text { FI}$, $\alpha _\text { IF}$ and $\alpha _\text { II}$, is given in [54, 55, 86, 87, 89]. It has the general form

C.6 $$\begin{aligned} A_{ab}^K(x,\{\alpha _\text {dip}\})&= A_{ab}^{K_{}^t}(x,\alpha _\text { FI},\alpha _\text { IF})+A_{ab}^{\tilde{K}}(x,\alpha _\text { II}). \end{aligned}$$

The superscripts indicate the general functional form of the contribution. In case of the $\alpha _\text { FI}$ and $\alpha _\text { IF}$ depedence the existing plus distributions are modified in the $K_{}^t$ terms as follows

C.7 $$\begin{aligned} \left( ..\right) _+ \rightarrow \left( ..\right) _{1-\alpha _\text { FI}} \end{aligned}$$

with

C.8 $$\begin{aligned} \int \limits _0^1\mathrm{d}x\; f(x) [g(x)]_{1-\alpha _\text { FI}}&= \int \limits _{1-\alpha _\text { FI}}^1\mathrm{d}x\; g(x) [f(x)-f(1)]. \end{aligned}$$

As the singular point sits at $x=1$ this modification amouts to a finite contribution. The $\alpha _\text { IF}$ on the other hand leads to finite regular functional contribution in the $A_{ab}^{K_{}^t}$ term given in [87, 89].

Lastly, the $\alpha _\text { II}$ dependence reads [55]

C.9 $$\begin{aligned} A_{ab}^{\tilde{K}}(x,\alpha _\text { II}) = \log \frac{\alpha _\text { II}}{1-x}, \end{aligned}$$

except when $a=b=\gamma$, in which case it is zero as the original $\tilde{K}_{\gamma \gamma }$ term.

## Footnotes

1 To be precise the CT14nlo and CT14qed_inc_proton PDF sets interfaced through Lhapdf [79] 6.2.1 are used.

2 For advances for NLO calculations with initial state massive particles see [44, 81, 82].

3 This assumption also proves to be correct for on-shell *W* production at a 100 TeV collider.
